# General Spin Restricted Open-Shell Configuration Interaction Singles (GS-ROCIS): Implementation of Spin–Orbit Coupling and Zeeman Operators for Calculation of Optical and X‑ray Absorption and Magnetic Circular Dichroism Spectra of Magnetically Coupled Transition Metal Systems

**DOI:** 10.1021/acs.jpca.5c05086

**Published:** 2025-09-30

**Authors:** Tiago Leyser da Costa Gouveia, Lucas Lang, Dimitrios Maganas, Frank Neese

**Affiliations:** † 28314 Max-Planck-Institut für Kohlenforschung, Kaiser-Wilheim-Platz 1, Mülheim an der Ruhr 45470, Germany; ‡ 26524 Technische Universität Berlin, Institut für Chemie, Theoretische Chemie/Quantenchemie, Sekr. C7, Straße des 17. Juni 135, 10623, Berlin, Germany

## Abstract

In this paper we present the theory and implementation of the spin–orbit coupling and Zeeman operators in the context of quasi-degenerate perturbation theory into the general spin restricted open-shell configuration interaction singles method. The implementation of the mentioned operators allows for the calculation of magnetic circularly polarized dichroism (MCD), L-edge X-ray absorption spectra (XAS) and X-ray magnetic circularly polarized dichroism (XMCD) spectra. The method was tested on calculating the MCD spectra of isostructural complexes [LCr^III^(PyA)_3_Ni^II^]^2+^, [LCr^III^(PyA)_3_Zn^II^]^2+^and [LGa^III^(PyA)_3_Ni^II^]^2+^, with *L* = 1,4,7-trimethyl-1,4,7-triazacyclonanane and PyA^–^ is the monoanion of pyridine-2-aldozime, where it correctly predicts the MCD signs of the lower optical transition of [LCr^III^(PyA)_3_Ni^II^]^2+^and [LGa^III^(PyA)_3_Ni^II^]^2+^. The capabilities of the method in computing L-edge XAS and XMCD spectra were tested on the model complexes [Cu­(H_2_O)_6_]^2+^ and [Cu_2_(OAc)_4_(H_2_O)_2_], where it correctly calculates the L_2,3_-edge absorption and XMCD spectra, as well as on the antiferromagnetically coupled Cu–Fe dimer [(F_8_TPP)­Fe­(μ-O)­Cu­(TMPA)]^+^, where it correctly predicts the signs of the L_2_ and L_3_ edges of the Cu XMCD spectrum. To further illustrate the applicability of the method, the more complex L_2,3_-edge XAS and XMCD spectra of thiolate Fe complexes were also calculated.

## Graphical abstract

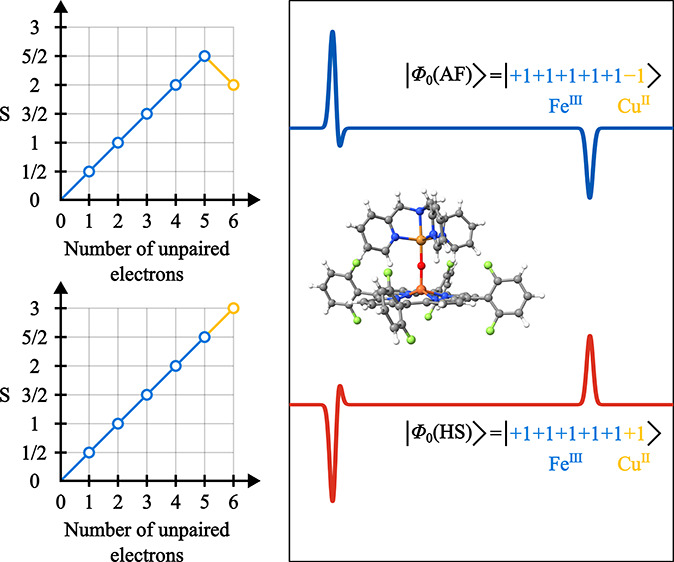

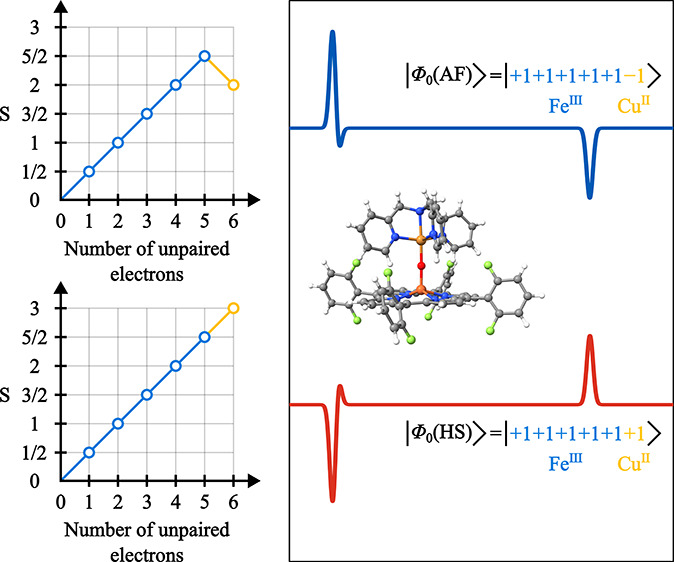

## Introduction

X-ray spectroscopy techniques such as X-ray absorption (XAS), emission (XES) and resonant emission (RXES) are able to probe the electronic transitions involving core electrons of individual nuclei in a sample, providing local, element-specific electronic structure information, making these techniques an essential tool in the fields of molecular,^1–7^ biological^8–15^ and solid-state^16–22^ chemistry. Furthermore, by combining magnetic circular dichroism (MCD) with X-ray spectroscopies in an XMCD experiment, site-resolved insights into the electronic and magnetic properties of the metal centers can be obtained, with exceptional sensitivity to both spin and orbital magnetic moments.^23^ By directly probing the local magnetic asymmetry in the absorption of circularly polarized X-rays, XMCD enables the disentanglement of spin and orbital contributions, even in complex antiferromagnetic systems. It overcomes the limitations of conventional magnetometry techniques, making it an indispensable method for investigating the intricate physics of antiferromagnetically coupled systems.^24–27^

However, due to the high density of many-particle states, or the dependence of experimental conditions such as temperature and magnetic field that may be probed in such X-ray spectroscopy techniques, the extraction of structural and/or electronic information directly from the experimental spectra is not always achievable. Consequently, theoretical methods play a key role in the interpretation of the obtained experimental spectra.^28^

In the context of transition metal complexes, a theoretical method needs to properly describe ligand-field splittings, metal–ligand covalency and multiplet effects in both ground and core-excited states. A further major complication arises when the investigated systems contain antiferromagnetically coupled open-shell centers, inevitably leading to even more complex multiplet problems.

To properly deal with the multiplet effects employing wave function based methods, one can resort to multideterminantal or multiconfigurational methods, capable of describing the spin eigenfunctions that can be formed from a given electronic configuration. In this context protocols that are based on the complete active space configuration interaction methods (CASCI) in conjunction with N-electron valence second order perturbation theory^29^ (NEVPT2) or restricted active space self-consistent field (RASSCF) together with restricted active space perturbation theory^30^ (RASPT2), namely CASCI/NEVPT2 and RASSCF/RASPT2, as well as multireference configuration interaction (MRCI) or multireference equation of motion coupled cluster (MREOM-CC) have been successfully employed in calculating XAS,^31–35^ resonant inelastic X-ray scattering (RIXS)^36–38^ and XMCD^39,40^ spectra. The downside of such methods is their typically high computational cost, which limits their applicability to a narrow range of small to medium-sized molecular systems. Hence, in practical application on large systems such as the cofactors of metalloenzymes, methods with lower computational demands, but that are still able to capture aspects of the antiferromagnetic coupling and multiplet effects are commonly used.

In the density functional theory formalism (DFT), one of the most popular approaches in dealing with low-spin states is to break the spin symmetry of the system,^41–45^ leading to the popular broken-symmetry DFT (BS-DFT) method. The electronic core excitations can then be obtained by means of time-dependent DFT (TD-DFT) calculations realized on top of the broken-symmetry determinant.^46–48^ Although popular, BS-DFT results in a very crude approximation to antiferromagnetic coupling,^49,50^ and in systems with complex spin coupling situations, the possibility of several broken-symmetry solutions makes it difficult to assign a specific spin coupling situation for the system being studied.^9,51^

The low-spin states can also be approach by the spin-flip family of methods,^52^ where one obtain the lower spin states by spin-flip excitations over a high-spin reference. This approach has been successfully applied within the framework of configuration interaction (CI),^53–56^ equation-of-motion coupled-cluster (EOM-CC)^57–59^ and TD-DFT.^60–63^ One of the limitations of many spin-flip approaches is the spin incompleteness of the excitation space, potentially leading to the artificial mixing of states of different spin multiplicities. This limitation can be solved by restoring the spin completeness of the excitation space by including higher excitations. Furthermore, multiple spin-flip excitations can be accounted for in the form of the restricted active space spin-flip (RAS-SF) methods.^64,65^ A more detailed review of the spin-flip approaches is given by Casanova and Krylov.^52^

Instead of starting from a high-spin reference to obtain the low-spin states, which requires the spin adaptation to be considered after the generation of the excitation space, one can start from a spin-adapted low-spin reference and realize the excitation problem conserving the spin adaptation. An example of this approach is the restricted open-shell configuration interaction singles (ROCIS) method,^66,67^ where from a restricted open-shell Hartree–Fock (ROHF) reference, classes of excited configuration state functions (CSFs) are created while maintaining the spin adaptation through the whole procedure. In particular, ROCIS and its usage with pair natural orbitals (PNO-ROCIS)^68^ as well as their DFT parametrized versions (ROCIS/DFT and PNO-ROCIS/DFT) have been used to compute a variety of XAS^69–78^ and valence-to-core resonance X-ray emission spectra (VtC-RXES)^79^ of molecules and solids.

While originally only applicable to high-spin references, the method was recently generalized to references of any spin coupling situation by the development of the CSF-ROHF method,^80^ capable of providing Hartree–Fock quality wave functions for a given CSF and the general-spin ROCIS (GS-ROCIS).^81^ These new developments by themselves allows for the computation of K-edge XAS spectra, however for their application to L- and M-edge XAS, XES, RIXS and XMCD, a proper treatment of spin–orbit coupling (SOC) is needed.

The most complete description of relativistic effects, of which SOC is one of, would require the use of the four-component Dirac-Coulomb-Breit Hamiltonian.^82^ However, for practical calculations the four-component approach is computationally expensive and, therefore, further approximations are used. By means of the Breit-Pauli approximation,^83–86^ it is possible to reduce the four-component relativistic Hamiltonian to a two-component form in which the SOC operator has a one- and two-electron part. This form of the SOC operator can be further approximated by treating the two-electron part in a mean-field approach, resulting in an effective one-electron SOC operator.^87,88^

Although the inclusion of SOC variationally is possible,^89^ the most common approach is to consider SOC and other spin-dependent operators only at the many-electron level, in a two-step approach where first the nonrelativistic many-particle wave functions are calculated and in the second step the spin-dependent operators are diagonalized in the many-particle basis. This two-step approach has been applied in several instances such as in the restricted active space state interaction (RASSI) method,^90,91^ quasi degenerate perturbation theory (QDPT) in the context of MRCI,^92^ ROCIS^67^ and density matrix renormalization group (DMRG),^93^ EOM-CC^94–96^ among others.

Following the two-step approach, in this work, we present the implementation of SOC and Zeeman effects on top of the GS-ROCIS wave function on the basis of quasi-degenerate perturbation theory (QDPT). The implementation is then tested in calculations of optical MCD as well as metal L_2,3_-edge X-ray absorption and XMCD spectra of different metal dimers with increasing complexity of the spin coupling situation. It is demonstrated that the intrinsic spin sensitivity of MCD and XMCD is instrumental for probing the spectroscopic properties of ferromagnetic (FM) and antiferromagnetic (AFM) spin coupling systems.

## Theory

### General Spin ROCIS

We start with a brief description the GS-ROCIS method.^81^ Starting from a reference CSF constructed in the genealogical coupling scheme,^97^ four classes of excited CSFs are defined according to the occupation number of the orbitals involved in the excitation. These classes are excitations from a doubly occupied molecular orbital (DOMO) to a singly occupied molecular orbital (SOMO) (|Φ_*i*_^*t*^⟩), excitations from a SOMO to a virtual molecular orbital (VMO) (*|*Φ_t_^a^⟩), DOMO to VMO excitations (|Φ_*i*_^a^⟩) and DOMO to VMO excitations coupled with SOMO to SOMO excitations (|Φ_ui_^at^⟩). Throughout the text, *i*, *j*, *k*, *l* indices represent DOMOs, *t*, *u*, *v*, *w* represent SOMOs, *a*, *b*, *c*, *d* indices represent VMOs and *p*, *q*, *r*, *s* indices represent generic molecular orbitals.

This way, the GS-ROCIS wave function takes the form of the linear combination bellow.

1 $$\Psi_{GS-ROCIS}=c_{0}|\Phi_{0}\rangle +\sum_{i,t}c_{i}^{t}|\Phi_{i}^{t}\rangle +\sum_{t,a}c_{t}^{a}|\Phi_{t}^{a}\rangle +\sum_{i,a}c_{i}^{a}|\Phi_{i}^{a}\rangle +\sum_{i,t,a,u\neq t}c_{ui}^{at}|\Phi_{ui}^{at}\rangle$$

The GS-ROCIS problem is then the determination of the expansion coefficients c_q_^p^, which is achieved by the diagonalization of the Born–Oppenheimer Hamiltonian ( ${Ĥ}_{BO}$ ) expressed in the basis of the CSFs. In this basis, the matrix elements are given by

2 $$\left\langle \Phi_{I}\left|{Ĥ}_{BO}\right|\Phi_{J}\right\rangle =\sum_{pq}h_{pq}\left\langle \Phi_{I}\left|{Ê}_{q}^{p}\right|\Phi_{J}\right\rangle +\frac{1}{2}\sum_{pqrs}\left(pq|rs\right)\left(\left\langle \Phi_{I}\left|{Ê}_{q}^{p}{Ê}_{s}^{r}\right|\Phi_{J}\right\rangle -\delta_{qr}\left\langle \Phi_{I}\left|{Ê}_{s}^{p}\right|\Phi_{J}\right\rangle \right)$$

where we make use of the singlet excitation operator ${Ê}_{q}^{p}={â}_{q\beta }^{†}{â}_{p\beta }+{â}_{q\beta }^{†}{â}_{p\beta }$ , with the ${â}_{p\sigma }^{†}$ and ${â}_{p\sigma }$ being the standard creation and annihilation operators, respectively, related to orbital *p* and spin function σ. The one electron integrals are represented by *h*_pq_, the two-electron integrals by (pq|rs), δ_qr_ is a Kronecker delta and Φ_*I*_ and Φ_*J*_ represent arbitrary CSFs.

As mentioned above, the CSFs used as the many-electron basis in GS-ROCIS are constructed following the genealogical coupling scheme, which allows for the efficient calculation of the $\left\langle \Phi_{I}\left|{Ê}_{q}^{p}\right|\Phi_{J}\right\rangle$ and $\left\langle \Phi_{I}\left|{Ê}_{q}^{p}{Ê}_{s}^{r}\right|\Phi_{J}\right\rangle$ matrix elements needed for the construction of ${Ĥ}_{BO}$ . The full description of the GS-ROCIS method can be found elsewhere.^81^

The above-described procedure generates the excited states with the same spin multiplicity as the reference CSF (*S* ^′^ = *S*). We may wish to also generate excited states with spin quantum numbers *S* ^′^ = *S* + 1 and *S* ^′^ = *S* – 1, since these are allowed to mix with the *S* ^′^ = *S* states through the spin–orbit coupling operator, as will be described later. The different spin multiplicity CSFs are generated from the reference CSF by performing orbital excitations, followed by the appropriate spin recoupling.

As an example, we take a reference CSF with 4 unpaired electrons and *S* = 1, represented by the branching diagram in Figure 1a, where the coupling of a SOMO with the previous one is represented by an upward (for parallel coupling) or downward (for antiparallel coupling) line. We can represent this CSF as a string of numbers, where a number 2 represents a DOMO, a number 0 a VMO and +1 and −1 represents SOMOs with parallel or antiparallel coupling, respectively. From this reference, the *S* ^′^ = *S* + 1 CSFs (Figure 1b) can be obtained from DOMO to SOMO excitations (|Φ_*i*_^*t*+^⟩), SOMO to VMO excitations (|Φ_*t*_^*a*+^⟩) and DOMO to VMO excitations (|Φ_*i*_^*a*+^⟩), where the superscript + indicates the increase in the spin multiplicity.

**Figure 1:**
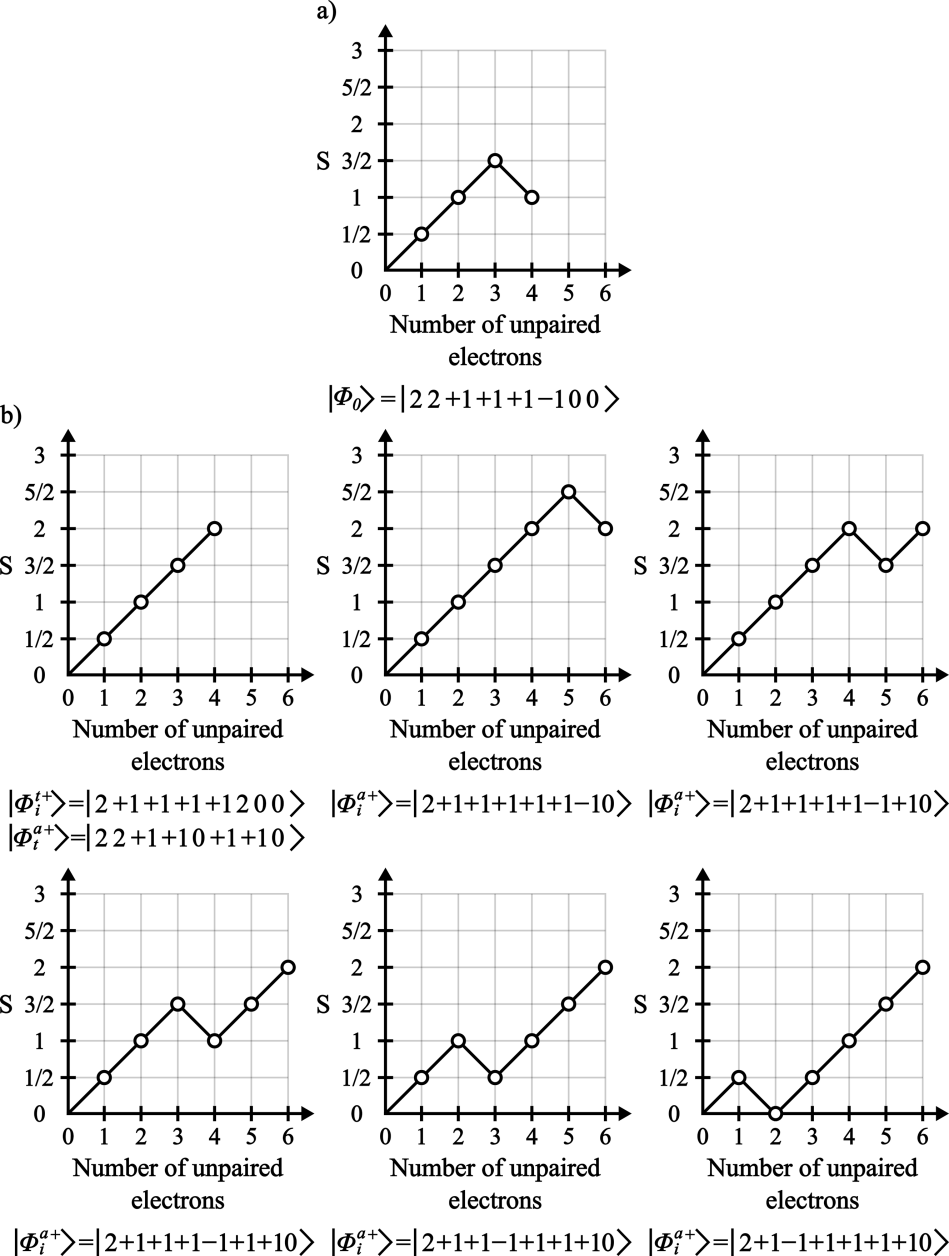
(a) Reference CSF branching diagram for a system with 4 unpaired electrons and *S* = 1. (b) Branching diagrams for the excited CSFs with *S* ^′^ = *S* + 1, generated from the reference CSF.

The *S* ^′^ = *S* – 1 CSFs can also be obtained from these orbital excitations (|Φ_*i*_^*t*–^⟩, |Φ_*t*_^*a*–^⟩ and |Φ_*i*_^*a*–^⟩) and from SOMO to SOMO excitations (|Φ_*t*_^*u*–^⟩), where the superscript—represents the lowering of the spin multiplicity. For this case, the orbital excitations allow for more than one spin coupling situation. The excitation space is then comprised of all possible branching diagrams for a given excitation as shown in Figure 2.

**Figure 2:**
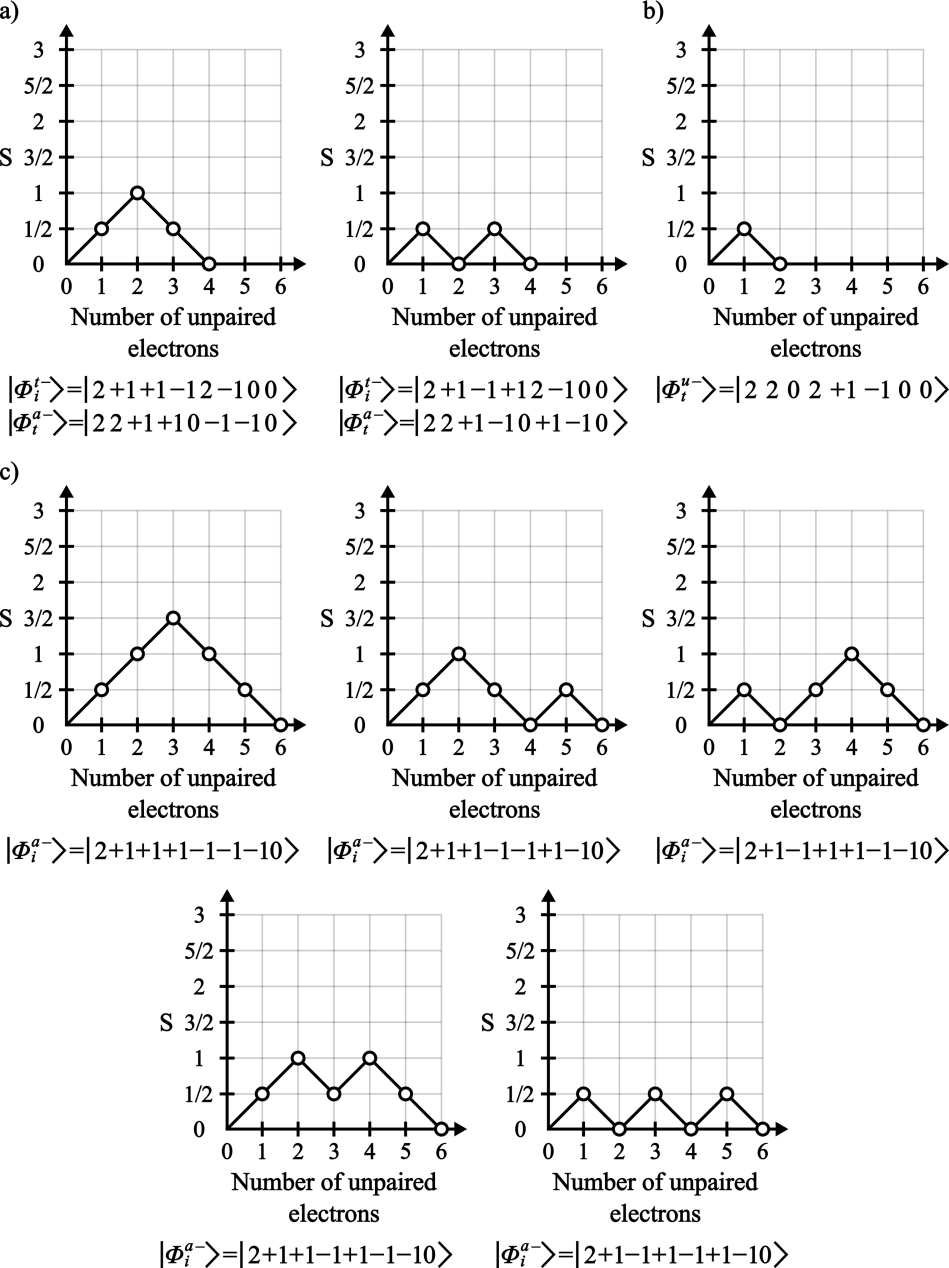
Branching diagrams representing all spin coupling situations for the (a) |Φ_*i*_^*t*–^⟩ and |Φ_*t*_^*a*–^⟩ excitation classes, (b) the |Φ_*t*_^*u*–^⟩ class and (c) the |Φ_*i*_^*a*–^⟩ class of CSFs of the *S* ^′^ = *S* – 1 GS-ROCIS problem.

Since the BO Hamiltonian does not mix CSFs with different spin multiplicities, we end up with three distinct CI problems that are solved independently. The resulting states are then used in constructing the spin–orbit Hamiltonian as described below.

### Quasi-Degenerate Perturbation Theory

The inclusion of spin-dependent properties in GS-ROCIS is introduced in the framework of quasi-degenerate perturbation theory (QDPT).^90,92^ In the QDPT framework, the spin-dependent operators are introduced as a perturbation to the ${Ĥ}_{BO}$ , resulting in the effective Hamiltonian

3 $$Ĥ={Ĥ}_{BO}+{Ĥ}_{SOC}+{Ĥ}_{SSC}+{Ĥ}_{Z}$$

With ${Ĥ}_{SOC}$ , ${Ĥ}_{SSC}$ and ${Ĥ}_{Z}$ being the spin–orbit coupling, spin–spin coupling and Zeeman operators, respectively. For the purposes of this paper, however, we will focus on the implementation of only ${Ĥ}_{SOC}$ and ${Ĥ}_{Z}$ .

The matrix elements of the effective Hamiltonian including SOC and Zeeman interactions in the basis of the nonrelativistic states obtained after the CI procedure takes the form

4 $$\left\langle \Psi_{I}^{S,M}\left|{Ĥ}_{BO}+{Ĥ}_{SOC}+{Ĥ}_{Z}\right|\Psi_{J}^{S^{\prime }{,M}^{\prime }}\right\rangle =\delta_{IJ}\delta_{SS^{\prime }}\delta_{MM^{\prime }}E_{I}^{\left(S\right)}+\left\langle \Psi_{I}^{S,M}\left|{Ĥ}_{SOC}+{Ĥ}_{Z}\right|\Psi_{J}^{S^{\prime }{,M}^{\prime }}\right\rangle$$

where Ψ_*I*_^S,M^ represent the wave function of state *I*, with total spin *S* and projection M and *E*_I_^(S)^ is the energy of state *I*, obtained from the solution of the preceding CI calculation. The diagonalization of the combined Hamiltonian matrix yields the spin–orbit coupled eigenstates and respective energies as linear combinations of the solutions of ${Ĥ}_{BO}$ with complex expansion coefficients.

### Spin–Orbit Coupling

In this work, we approximate the spin–orbit coupling (SOC) operator by the spin–orbit mean field (SOMF) operator,^87,88^ which is a triplet one-electron operator and can be written as

5 $${Ĥ}_{SOMF}=\sum_{m}^{-1,0,1}{\left(-1\right)}^{m}\sum_{pq}z_{pq}^{-m}{ŝ}_{pq}^{m}$$

where *z*_pq_^(0, ± 1)^ are the spherical tensor components and ${ŝ}_{pq}^{\left(0,\pm 1\right)}$ are the spin operators

6 $${ŝ}_{pq}^{\left(+1\right)}=-\frac{1}{\sqrt{2}}{â}_{p\alpha }^{†}{â}_{q\beta }$$

7 $${ŝ}_{pq}^{\left(0\right)}=\frac{1}{2}\left({â}_{p\alpha }^{†}{â}_{q\alpha }-{â}_{p\beta }^{†}{â}_{q\beta }\right)$$

8 $${ŝ}_{pq}^{\left(-1\right)}=\frac{1}{\sqrt{2}}{â}_{p\beta }^{†}{â}_{q\alpha }$$

which are the three components of the triplet single-excitation operator.^98^

Employing the Wigner–Eckart theorem, the matrix elements of ${Ĥ}_{SOMF}$ take the form

9 $$\left\langle \Psi_{I}^{S,M}\left|{Ĥ}_{SOMF}\right|\Psi_{J}^{S^{\prime }{,M}^{\prime }}\right\rangle =\sum_{m}^{-1,0,1}{\left(-1\right)}^{m}\left(\begin{array}{ll} S^{\prime } & 1\\ M^{\prime } & m \end{array}|\begin{array}{l} S\\ M \end{array}\right)\sum_{pq}z_{pq}^{-m}\left\langle \Psi_{I}^{S}∥{ŝ}_{pq}∥\Psi_{J}^{S^{\prime }}\right\rangle$$

where $\left(\begin{array}{cc} S^{\prime } & 1\\ M^{\prime } & m \end{array}|\begin{array}{c} S\\ M \end{array}\right)$ is a Clebsch–Gordan coefficient.

Expanding the states |Ψ_*I*_^*S*^⟩ and $|\Psi_{J}^{S^{\prime }}\rangle$ into the linear combination of CSFs we obtain

10 $$\left\langle \Psi_{I}^{S,M}\left|{Ĥ}_{SOMF}\right|\Psi_{J}^{S^{\prime }{,M}^{\prime }}\right\rangle =\sum_{m}^{-1,0,1}{\left(-1\right)}^{m}\left(\begin{array}{ll} S^{\prime } & 1\\ M^{\prime } & m \end{array}|\begin{array}{l} S\\ M \end{array}\right)\sum_{KL}c_{KI}^{S}c_{LJ}^{S^{\prime }}\sum_{pq}z_{pq}^{-m}\left\langle \Phi_{K}^{S}∥{ŝ}_{pq}∥\Phi_{L}^{S^{\prime }}\right\rangle$$

Hence, for the calculation of the matrix elements of the SOMF Hamiltonian the computation of the coupling coefficient reduced matrix elements $\left\langle \Phi_{K}^{S}∥{ŝ}_{pq}∥\Phi_{L}^{S^{\prime }}\right\rangle$ is needed. This is achieved in a similar fashion as the one-body coupling coefficients needed for the matrix elements of the BO Hamiltonian, with an efficient “tree-walk” like algorithm which is better described elsewhere.^99^ Also, in analogy to the one-body coupling coefficients, the $\left\langle \Phi_{K}^{S}∥{ŝ}_{pq}∥\Phi_{L}^{S^{\prime }}\right\rangle$ elements are dependent only on the p, q indices in relation to the SOMOs of a given CSF. This way, one can employ a prototyping scheme similar to the one used in solving the GS-ROCIS problem, where it is necessary to generate only the minimal number of CSFs that can represent the entire excitation space of the problem to be solved (Figure 3). For the specific case of GS-ROCIS, since the excitation classes involve at most one DOMO and one VMO, a prototype space consisting of 2 DOMOs, 2 VMOs and the required number of SOMOs is enough to generate all possible matrix elements needed.

**Figure 3:**
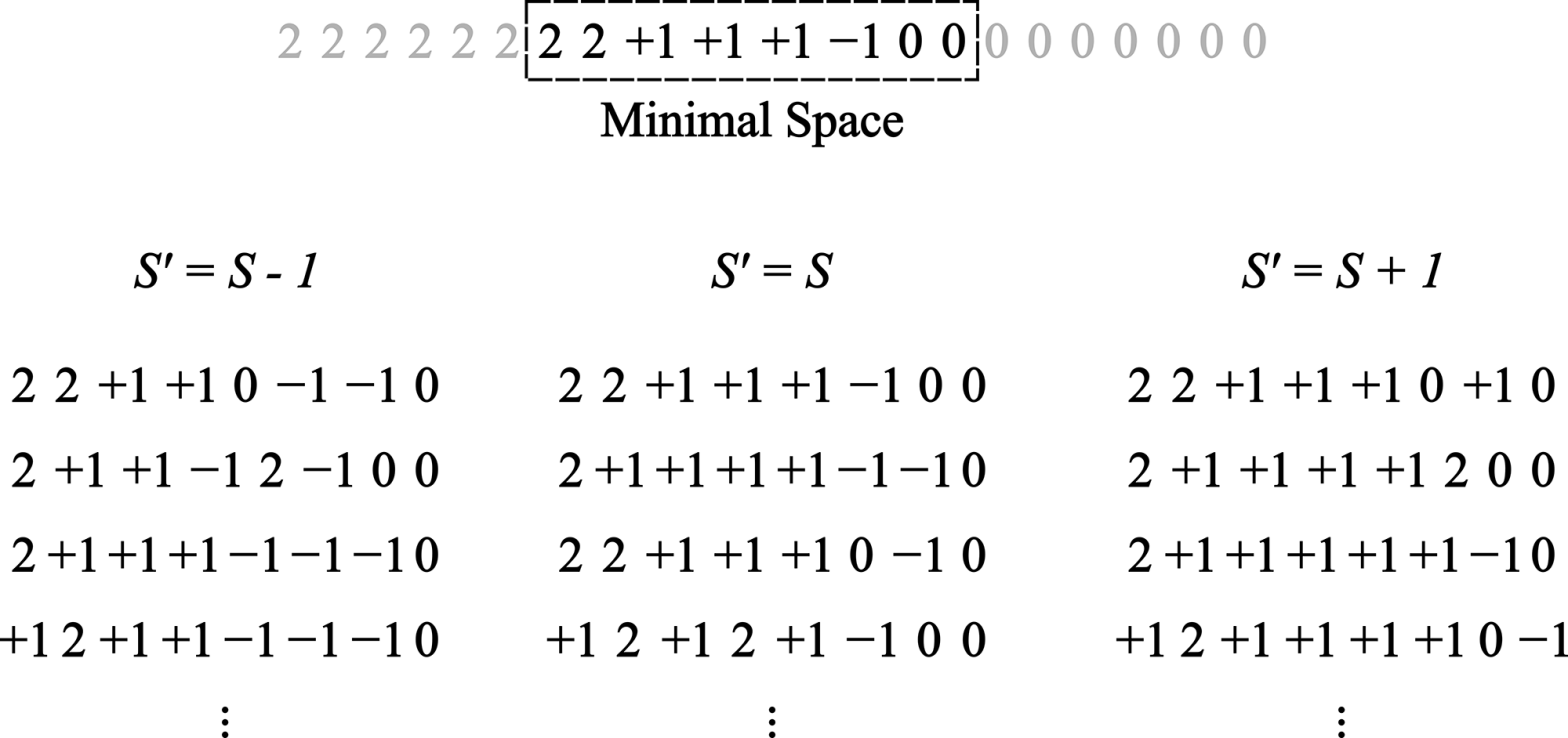
Representation of the minimal space needed to generate the prototype CSFs of the spin multiplicities used for the calculation of the coupling coefficient reduced matrix elements.

The full prototyping scheme then works as follows: first, the minimal space CSFs are generated for each of the spin multiplicities, resulting in distinct tree structures. Then, the matrix elements are calculated for the three spin blocks in a GS-ROCIS calculation, $\left\langle \Phi_{K}^{S}∥{ŝ}_{pq}∥\Phi_{L}^{S}\right\rangle$ , $\left\langle \Phi_{K}^{S-1}∥{ŝ}_{pq}∥\Phi_{L}^{S-1}\right\rangle$ and $\left\langle \Phi_{K}^{S+1}∥{ŝ}_{pq}∥\Phi_{L}^{S+1}\right\rangle$ , as well as for the two blocks of different spins $\left\langle \Phi_{K}^{S}∥{ŝ}_{pq}∥\Phi_{L}^{S-1}\right\rangle$ and $\left\langle \Phi_{K}^{S}∥{ŝ}_{pq}∥\Phi_{L}^{S+1}\right\rangle$ . The resulting elements are stored in memory and can be retrieved by specifying the multiplicity of bra and ket CSFs.

#### Zeeman Hamiltonian

The interaction of the system with an external homogeneous magnetic field is accounted for by the Zeeman Hamiltonian, which, in atomic units, is included in the form

11 $${Ĥ}_{Z}=\frac{1}{2}B\cdot \left(L+g_{e}S\right)$$

where *B* is the external magnetic field, *g*_e_ is the free-electron *g*-value, $L=\sum_{i}l_{i}$ is the total orbital angular momentum operator and $S=\sum_{i}s_{i}$ is the total spin angular momentum operator.

The Zeeman operator depends only on the *S* and *M* quantum numbers of the CSFs and, hence, can be calculated without need of the machinery described above for the SOC matrix elements.

### Relativistic Transition Densities and XMCD Signs

With the relativistic and magnetic field corrected states obtained from the diagonalization of the QDPT Hamiltonian, we can calculate the transition moment (*T*_PQ_) between states $|{\mathrm{\Psi ̃}}_{P}\rangle$ and $|{\mathrm{\Psi ̃}}_{Q}\rangle$ as

12 $$T_{PQ}=\left\langle {\mathrm{\Psi ̃}}_{P}\left|\mathrm{m̂}\right|{\mathrm{\Psi ̃}}_{Q}\right\rangle =\sum_{p,q}m_{pq}\left\langle {\mathrm{\Psi ̃}}_{P}\left|{Ê}_{q}^{p}\right|{\mathrm{\Psi ̃}}_{Q}\right\rangle =\sum_{I,J}c_{IP}^{*}c_{JQ}\sum_{p,q}m_{pq}\left\langle \Psi_{I}\left|{Ê}_{q}^{p}\right|\Psi_{J}\right\rangle$$

where *m*_pq_ is the one-electron integral of the respective one-electron operator m̂, which can be chosen to be the electric dipole operator, or to include higher moments such as the magnetic dipole, electric quadrupole or be the full field–matter interaction operator.^100^ The transition density $\left\langle {\mathrm{\Psi ̃}}_{P}\left|{Ê}_{q}^{p}\right|{\mathrm{\Psi ̃}}_{Q}\right\rangle$ is calculated by expanding the relativistic and magnetically corrected states into the linear combination of nonrelativistic states and using the precomputed nonrelativistic transition densities.

In the electric dipole approximation, the XMCD spectrum is calculated using the following equation^101,102^

13 $$\frac{\Delta \varepsilon }{E}=\gamma \sum_{PQ}N_{P}\left(\sum_{u,v,w}t_{uvw}l_{u}Im\left\langle {\mathrm{\Psi ̃}}_{P}\left|m_{v}\right|{\mathrm{\Psi ̃}}_{Q}\right\rangle \left\langle {\mathrm{\Psi ̃}}_{Q}\left|m_{w}\right|{\mathrm{\Psi ̃}}_{P}\right\rangle \right)$$

where Δε = ε_RCP_–ε_LCP_ is the difference in extinction coefficients of right and left circularly polarized light, *N*_P_ is the Boltzmann population of state $|{\mathrm{\Psi ̃}}_{P}\rangle$ , *m*_*v*_ is the electric transition dipole moment operator for Cartesian direction *v*, *t*_uvw_ = 1 for uvw = *xyz*, *zxy*, *yzx* and *t*_uvw_ = 0 otherwise, γ is a collection of constants and *l*_u_(*u* = *x*,*y*,*z*) is given by

14 l⃗=(sin⁡θsin⁡φ,sin⁡θcos⁡φ,cos⁡θ)

where θ and φ are Eulerian angles.

To account for the random orientation of the molecules in respect to the external magnetic field we average over all possible molecular orientations. The average XMCD intensities are then

15 $${\left(\frac{\Delta \varepsilon }{E}\right)}_{average}=\sum_{\mu \eta \tau }{\left(\frac{\Delta \varepsilon }{E}\right)}_{\mu \eta \tau }\sin\theta_{\tau }$$

where μ, η and τ denote the indices of the angular grid on which the summation is performed.

The full description of the ORCA implementation of the orientational averaged XMCD intensities can be found elsewhere.^100,102^

### Computational Details

All calculations were performed in a development version of the ORCA 6.1 suite of programs.^103–108^

Geometry optimizations of [LCr^III^(PyA)_3_Ni^II^]^2+^, [LGa^III^(PyA)_3_Ni^II^]^2+^, [Cu­(H_2_O)_6_]^2+^, [Cu_2_(OAc)_4_(H_2_O)_2_], [Fe­(SPh)_4_]^2–^, [Fe­(SDur)_4_]^−^ and [L_2_Fe^II,III^_2_S_2_]^3–^ were all performed for the high spin situations, employing unrestricted Kohn–Sham DFT (UKS) with the BP86 ^109,110^ functional and triple-ζ Def2-TZVP^111^ basis sets, together with the auxiliary basis Def2/J for the RI-J approximation.^112^ In all cases the Grimme’s dispersion correction D4 ^113–117^ was also used.

The molecular geometry of [(F_8_TPP)­Fe­(μ-O)­Cu­(TMPA)]^+^ was obtained from the crystal structure data from ref 118.

The wave functions used as reference for the GS-ROCIS calculations were obtained using the restricted open-shell Hartree–Fock (ROHF). For the antiferromagnetically coupled systems, the CSF-ROHF^80^ method was used to obtain the wave function for the CSF best describing the spin coupling situation. For the [Cu­(H_2_O)_6_]^2+^, [Cu_2_(OAc)_4_(H_2_O)_2_], [Fe­(SPh)_4_]^2–^, [Fe­(SDur)_4_]^−^ and [L_2_Fe^II,III^_2_S_2_]^3–^ complexes, the basis set x2c-TZVPall^119^ was used together with the x2c^120^ approach to include scalar relativistic effects. As for the [LCr^III^(PyA)_3_Ni^II^]^2+^, [LGa^III^(PyA)_3_Ni^II^]^2+^, the def2-SVP basis set was used.

For the MCD calculations, GS-ROCIS was employed together with the Def2-SVP basis set and the auxiliary basis Def2-SVP/C for the RI approximation.

GS-ROCIS calculations of L_2,3_-edge XAS and XMCD were performed with the inclusion of scalar relativistic effects via the x2c approach. The basis set x2c-TZVPall was used in conjunction with the auxiliary basis automatically generated via the AutoAux^121^ keyword in ORCA. In order to increase the speed of the calculations, the pair natural orbital approach^68^ was used (PNO-GS-ROCIS). The donor 2p orbitals were localized via the Pipek–Mezey^122^ localization scheme.

Calculated MCD, L_2,3_-edge absorption and XMCD spectra were obtained by processing the output files of the calculations with the orca_mapspc utility program, which applies a constant Gaussian broadening over the computed intensities. Following the usual conventions of the different techniques, MCD spectra are presented as the differential absorption of left circularly polarized (LCP) and right circularly polarized (RCP) light (LCP-RCP), while XMCD spectra are presented as RCP-LCP absorption.

## Results and Discussion

### MCD Spectra of [LCr^III^(PyA)_3_Ni^II^]^2+^, [LCr^III^(PyA)_3_Zn^II^]^2+^ and [LGa^III^(PyA)_3_Ni^II^]^2+^

As a proof of principle, we employed the GS-ROCIS method in the calculation of the MCD spectra of the complex [LCr^III^(PyA)_3_Ni^II^]^2+^, where *L* = 1,4,7-trimethyl-1,4,7-triazacyclononane and PyA^–^ is the monoanion of pyridine-2-aldozime, and the isostructural complexes [LCr^III^(PyA)_3_Zn^II^]^2+^, [LGa^III^(PyA)_3_Ni^II^]^2+^, where the Ni­(II) and Cr­(III) centers were substituted by the diamagnetic Zn­(II) and Ga­(III) respectively. In all complexes, the coordination environment of the metal centers can be described as trigonally distorted octahedrons, which can be approximated as belonging to the *C*_3_ point group.

The [LCr^III^(PyA)_3_Zn^II^]^2+^ complex is characterized as a $S=\frac{3}{2}$ ground state with the Cr­(III) having a 3d^3^ electronic configuration, while the [LGa^III^(PyA)_3_Ni^II^]^2+^ complex is characterized as *S* = 1 ground state, with the Ni­(II) center having a 3d^8^ electronic configuration. The [LCr^III^(PyA)_3_Ni^II^]^2+^ complex (Figure 4a) is characterized by magnetic susceptibility to have antiferromagnetic interaction between the Cr­(III) and Ni­(II) centers, with an exchange coupling constant *J* = −8.4 cm^–1^, resulting in a $S=\frac{1}{2}$ ground state.^123^

**Figure 4:**
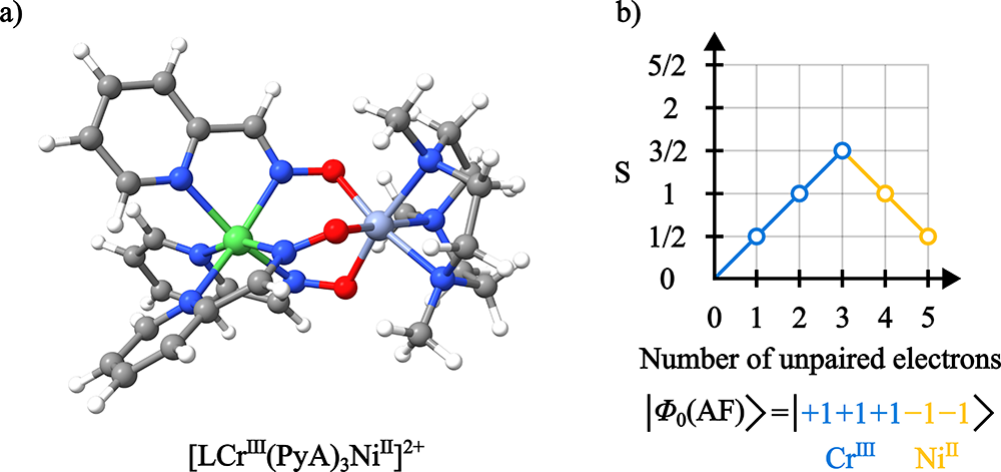
(a) Molecular structure of [LCr^III^(PyA)_3_Ni^II^]^2+^. (b) Branching diagram representative of the antiferromagnetic coupling CSF between the Cr and Ni centers.

Absorption and MCD spectra of the isostructural complexes were measured by Piligkos et al.^123^ at a temperature of 5K in a magnetic field of 3T. For each complex a simultaneous Gaussian deconvolution of the electronic absorption and MCD spectra was performed for the attribution of the observed electronic transitions.

The experimental attribution of the electronic transitions observed in the MCD spectrum of [LCr^III^(PyA)_3_Zn^II^]^2+^ are presented in Table 1, together with the calculated states obtained with GS-ROCIS. We recall that in approximate octahedral (∼*O*_h_ symmetry) coordination environment a d^3^ system possess a ^4^ *A*_2*g*_(^4^ *A* in *C*_3_ symmetry) ground state corresponding to the *t*_2g_^3^ *e*_*g*_^0^ electron configuration. Single electron *t*_2g_ → *e*_g_ excitations promoting one electron to the *e*_g_ shell result in a *t*_2g_^2^ *e*_*g*_^1^ electron configuration. This gives rise to a ^4^ *T*_2*g*_+^4^ *T*_1*g*_ excited state manifold, arising from the parent atomic ^4^ *F* term. As shown in Table 1, the calculated energy separation between the ^4^ *T*_2*g*_ and ^4^ *T*_1*g*_ excited states are overestimated by approximately 7300 cm^–1^ (0.90 eV) relative to experiment. This discrepancy can be traced to a fundamental limitation in the treatment of electron correlation within GS-ROCIS. In fact, double electron *t*_2*g*_→*e*_*g*_ excitations result in a *t*_2g_^1^ *e*_g_^2^ electron configuration, which give rise to a ^4^ *T*_1*g*_ state, originating from the corresponding atomic ^4^ *P* term.^124^ Typically these doubly excited states lying in close proximity with the same symmetry singly excited states mix strongly through the CI process. However, in the GS-ROCIS framework, double excitations are not included, leading to the absence of this mixing. As a result, the ^4^ *T*_1*g*_(^4^ *F*) state calculated with GS-ROCIS is artificially shifted to higher energies.

**Table 1:** Comparison of Calculated Transition Energies Obtained with GS-ROCIS to Experimentally Determined^123^ Transition Energies (in cm^–1^) of the [LCr^III^(PyA)_3_Zn^II^]^2+^ Dimer^a^

	state	energy (GS-ROCIS)	energy (experiment)
[LCr^III^(PyA)_3_Zn^II^]^2+^	^2^ *E*(^2^ *T*_1*g*_)	10,362	14,000–17,500
		10,491	
	^2^ *A*(^2^ *T*_1*g*_)	10,990	
	^4^ *A*(^4^ *T*_2*g*_)	17,948	18,450
	^4^ *E*(^4^ *T*_2*g*_)	18,807	19,490
		18,833	
	^2^ *E*(^2^ *E*_*g*_)	19,732	14,000–17,500
		19,900	
	^2^ *A*(^2^ *T*_2*g*_)	21,671	20,700
	^2^ *E*(^2^ *T*_2*g*_)	21,917	21,140
		22,140	
	^4^ *A*(^4^ *T*_1*g*_)	29,910	23,200
	^4^ *E*(^4^ *T*_1*g*_)	30,838	24,150
		30,856	

a State labeling is according to *C*_3_ and *O*_h_ (in parentheses) symmetries.

GS-ROCIS is also able to calculate the right energy ordering of the spin-flip states ^2^ *E*(^2^ *T*_1g_), ^2^ *A*(^2^ *T*_1g_), ^2^ *E*(^2^ *T*_2g_) and ^2^ *A*(^2^ *T*_2g_) which are described by the |Φ_*t*_^*u*–^⟩ class of excited CSFs with *t*≠*u*, where an electron is promoted between different singly occupied orbitals. In contrast, states corresponding to the |Φ_*t*_^*u*–^⟩ class of CSFs with *t* = *u* such as the ^2^ *E*(^2^ *E*_g_) state are less accurately reproduced. This reflects the absence of dynamic correlation of the method, which is particularly critical in spin-flip excitations. These transitions involve a delicate balance of correlation energies between initial and final states, and their accurate prediction requires a more sophisticated handling of differential dynamic correlation effects than is currently available in GS-ROCIS. These limitations underscore the need for future methodological developments focused on the inclusion of dynamic correlation effects, particularly for cases involving spin-flip transitions and double excitations.

Turning to the [LGa^III^(PyA)_3_Ni^II^]^2+^ dimer, the Gaussian deconvolution of its MCD spectrum shows four bands with negative MCD intensity, centered at 11,900, 12,900, 19,000, and 20,500 cm^–1^, which were attributed by the authors as the transitions between the ^3^ *A* ground state and the ^3^ *A*(^3^ *T*_2*g*_), ^3^ *E*(^3^ *T*_2*g*_), ^3^ *A*(^3^ *T*_1*g*_) and ^3^ *E*(^3^ *T*_1*g*_) states of the Ni­(II) center, respectively. Again, GS-ROCIS correctly predicts the energy ordering of the states (Table 2), but overestimates the energy difference between the two sets of states by ca. 7310 cm^–1^ (0.91 eV). Analogously with the situation of the excited ^4^ *T*_1*g*_ states of [LCr^III^(PyA)_3_Zn^II^]^2+^, the energy of the states arising from the ^3^ *T*_1*g*_ term of the Ni­(II) center are overestimated due to the absence of the higher energy ^3^ *T*_1*g*_ state resulting from double excitations, which would have a strong CI mixing with singly excited ^3^ *T*_1*g*_ state.

**Table 2:** Comparison of Calculated Transition Energies Obtained with GS-ROCIS to Experimentally Determined^123^ Transition Energies (in cm^–1^) of the [LGa^III^(PyA)_3_Ni^II^]^2+^ and [LCr^III^(PyA)_3_Ni^II^]^2+^ Dimers^b^

	state^a^	energy (GS-ROCIS)	energy (experiment)
[LGa^III^(PyA)_3_Ni^II^]^2+^	^3^ *A*(^3^ *T*_2*g*_)	11,579	11,900
	^3^ *E*(^3^ *T*_2*g*_)	11,854	12,900
		12,163	
	^3^ *A*(^3^ *T*_1*g*_)	25,789	19,000
	^3^ *E*(^3^ *T*_1*g*_)	26,873	20,500
		26,925	
[LCr^III^(PyA)_3_Ni^II^]^2+^	^3^ *A*(^3^ *T*_2*g*_)	15,355	11,900
	^3^ *E*(^3^ *T*_2*g*_)	15,654	12,900
		15,946	

a The state labeling for the [LCr^III^(PyA)_3_Ni^II^]^2+^ dimer is related to the local multiplicity (*S*_Ni_ = 1) of Ni­(II). The total multiplicity of the system is treated as $S=\frac{1}{2}$ .

b State labeling is according to *C*_3_ and *O*_h_ (in parentheses) symmetries.

The MCD spectrum of [LCr^III^(PyA)_3_Ni^II^]^2+^ can be described as a superposition of the spectra of the diamagnetically substituted dimers [LCr^III^(PyA)_3_Zn^II^]^2+^ (Figure S1) and [LGa^III^(PyA)_3_Ni^II^]^2+^ (Figure S2), with the distinction that the bands centered at 11,900 and 12,900 cm^–1^ have positive MCD intensity, which is presented as evidence that MCD can provide an experimental means of identifying the minority spin species in an coupled dimer.^123^

To probe the electronic states of the coupled dimer [LCr^III^(PyA)_3_Ni^II^]^2+^, a GS-ROCIS calculation is performed on top of the converged CSF-ROHF wave function of [LCr^III^(PyA)_3_Ni^II^]^2+^ in an open-shell doublet ( $S=\frac{1}{2}$ ) ground state represented by the CSF shown in Figure 4b. The resulting three lowest lying states are correspondent to the ^3^ *A*(^3^ *T*_2*g*_) and ^3^ *E*(^3^ *T*_2*g*_) excited Ni­(II) states (Table 2). The calculated MCD spectra of both [LGa^III^(PyA)_3_Ni^II^]^2+^ and [LCr^III^(PyA)_3_Ni^II^]^2+^ is shown in Figure 5, where it is readily observable that the correct MCD intensity sign is obtained.

**Figure 5:**
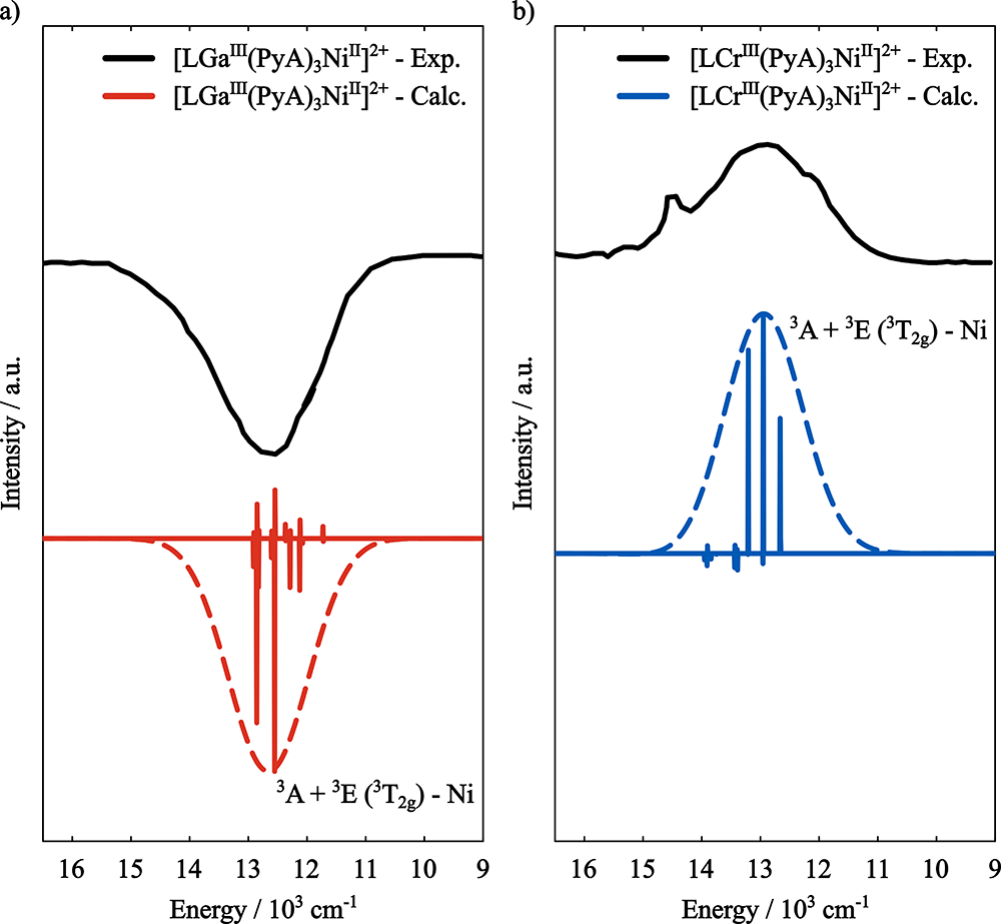
(a) Experimental and GS-ROCIS calculated MCD spectra of (a) [LGa^III^(PyA)_3_Ni^II^]^2+^ (shift = 500 cm^–1^) and of (b) [LCr^III^(PyA)_3_Ni^II^]^2+^ (shift = −2700 cm^–1^). Both spectra were calculated with B = 3T at a temperature of 5 K and a Gaussian broadening of 1000 cm^–1^ was applied. Experimental spectra were digitized from ref 123.

Hence, we showed that GS-ROCIS can provide a qualitative description of the electronic excited states probed in MCD spectroscopy for complexes containing both interacting and noninteracting metal centers. It is also able to provide the correct MCD sign for the antiferromagnetically coupled [LCr^III^(PyA)_3_Ni^II^]^2+^ dimer, demonstrating that the minority spin species in an antiferromagnetically coupled dimer will reverse the signs that the MCD bands would have in the absence of the second magnetic ion.

### L_2,3_-Edge XAS and XMCD of Model Cu Systems

[Cu­(H_2_O)_6_]^2+^

In order to better illustrate the obtained results for dimers containing magnetically coupled Cu centers, we first describe the electronic structure of the model [Cu­(H_2_O)_6_]^2+^ mononuclear complex (Figure 6a) and briefly discuss its L_2,3_-edge absorption and XMCD spectra.

**Figure 6:**
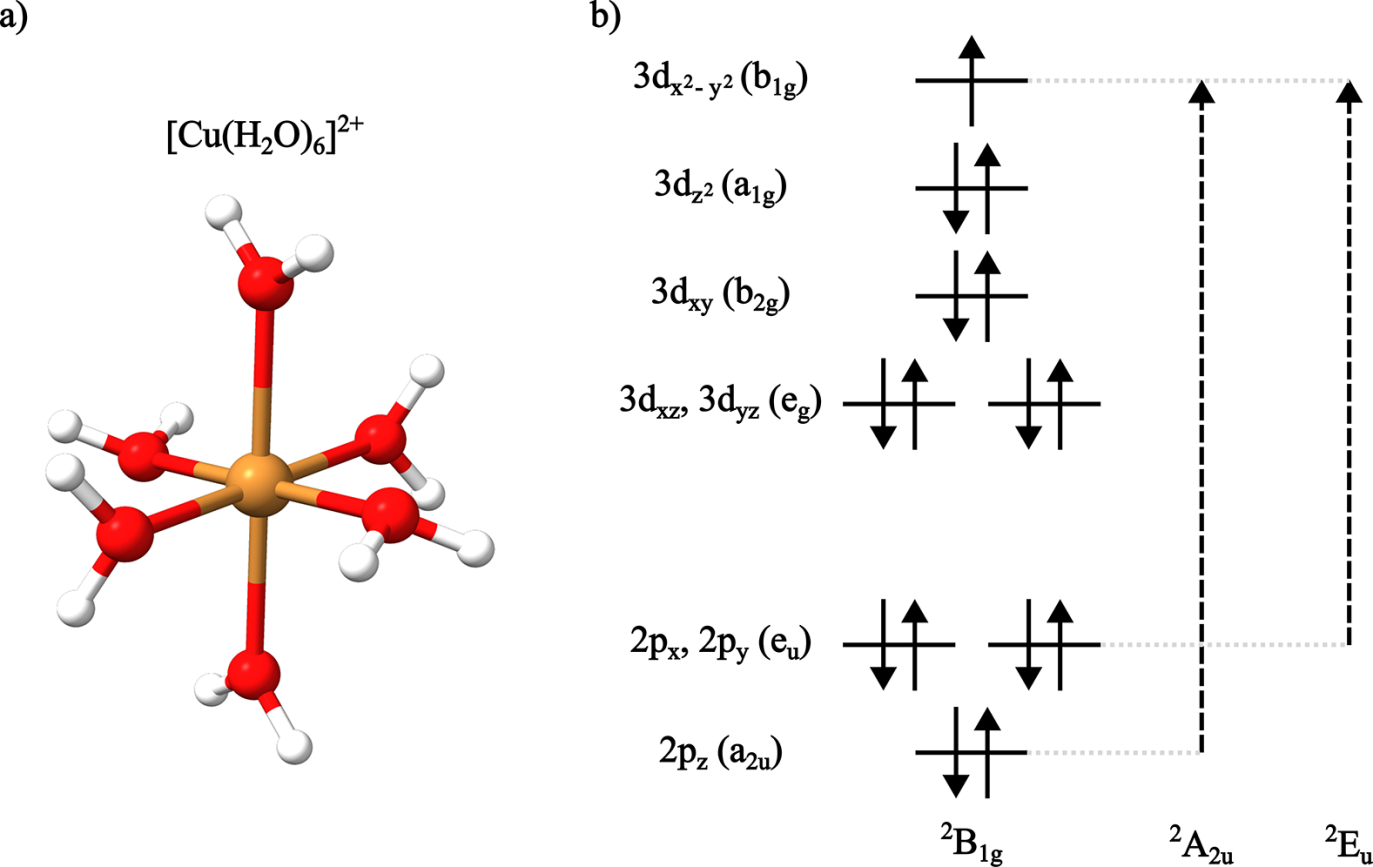
(a) Molecular geometry of [Cu­(H_2_O)_6_]^2+^. (b) Orbital diagram for the Cu­(II) center of [Cu­(H_2_O)_6_]^2+^ with *D*_4h_ symmetry labels for both orbitals, ground and excited states.

The Cu­(II) center in [Cu­(H_2_O)_6_]^2+^ has a *d* ^9^ electronic configuration in an approximate *D*_4*h*_ symmetry, resulting from a tetragonal distortion due to the Jahn–Teller effect. In this symmetry, the SOMO $d_{x^{2}-y^{2}}$ transforms as a *b*_1*g*_ representation, and the 2*p* core orbitals transform as *e*_*u*_ and *a*_2*u*_ (*p*_*x*_, *p*_*y*_ and *p*_*z*_ respectively). The ground state is then a ^2^ *B*_1*g*_, while the excited states arising from single excitations from the 2p orbitals are ^2^ *E*_*u*_ and ^2^ *A*_2*u*_ (Figure 6b and Table S1). Taking into account the effects of spin–orbit coupling resulting from the hole created in the 2*p* shell, the ^2^ *E*_*u*_ state splits into *E*_3_^′^(Γ_7_) and *E*_2_^′^(Γ_6_) states and ^2^ *A*_2*u*_ becomes an *E*_2_^′^(Γ_6_), while the ^2^ *B*_1*g*_ ground state becomes a *E*_3_^′^(Γ_7_) where the new labels are with respect to the *D*_4_^′^ double group (Figure 7a).

**Figure 7:**
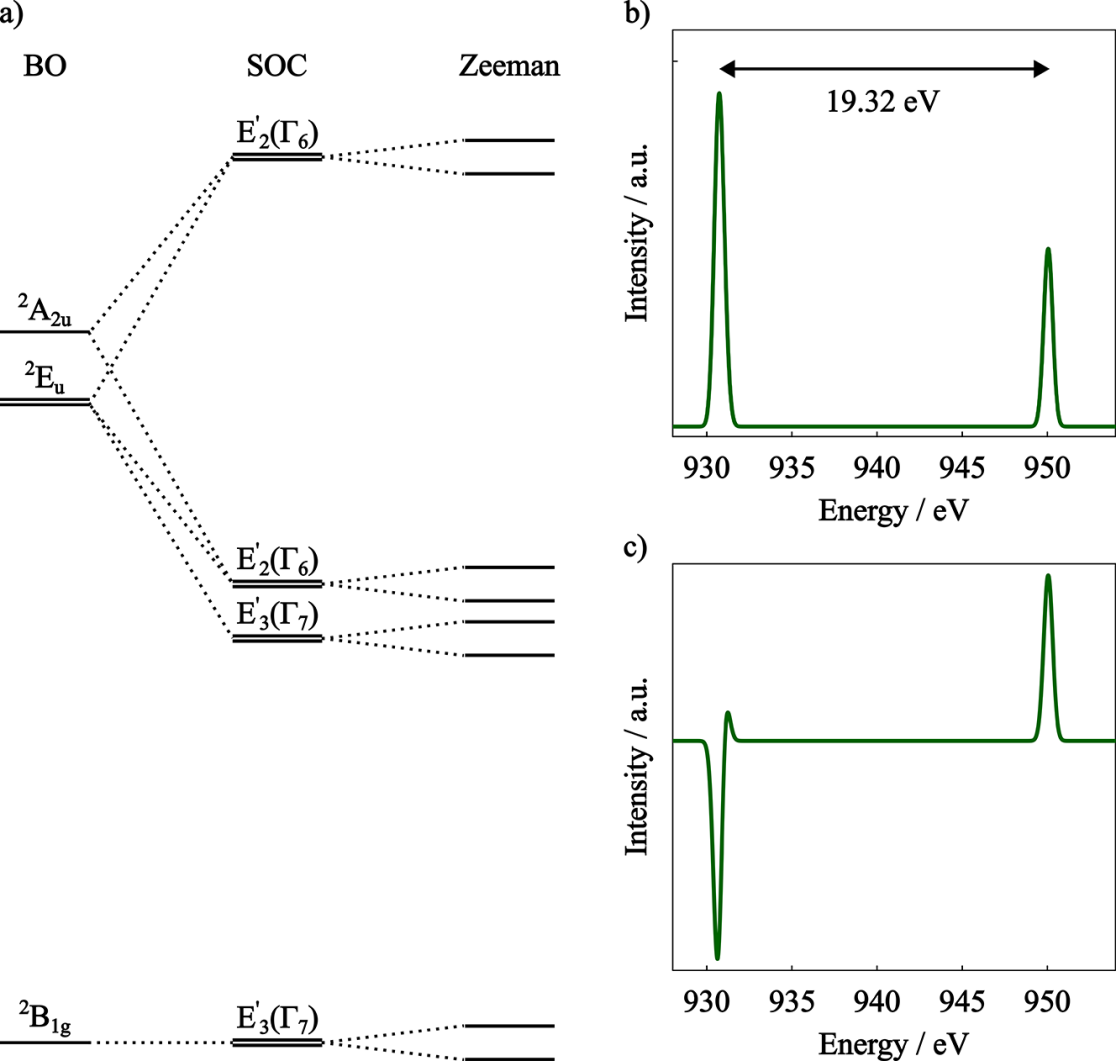
(a) [Cu­(H_2_O)_6_]^2+^ electronic states diagram with spin–orbit coupling (SOC) and Zeeman effects (not to scale). (b) Calculated L_2,3_-edge absorption spectrum and (c) XMCD (magnetic field of 3T and 4K temperature) of [Cu­(H_2_O)_6_]^2+^ within the electric dipole approximation. Basis set: x2c-TZVPall, Gaussian broadening: 0.62 eV.

The energy and composition of the obtained SOC corrected excited states are presented in Table S2 and the calculated L_2,3_-edge absorption spectrum is shown in Figure 7b. Due to the fully occupied 3*d* shell of the excited states, the calculated spectrum exhibits little structure, with only two features, the L_3_ and L_2_ edges, with an SOC induced splitting of 19.32 eV between the features. These results are in line with experimental results obtained for Cu­(II) monomer complexes.^125^

By applying an external magnetic field, the electronic states are subjected to the electronic Zeeman effect, which causes the splitting of the *E*_2_^′^(Γ_6_) and *E*_3_^′^(Γ_7_) states, as shown in Figure 7a. This splitting results in the calculated XMCD spectrum of [Cu­(H_2_O)_6_]^2+^ (Figure 7c) where now the L_3_ and L_2_ edges have opposite signs, the L_3_ absorbing more LCP than RCP radiation, while the L_2_ presenting the opposite behavior. This difference in sign is also observed in experimental XMCD spectra of Cu­(II) complexes.^10^

With this model system, it was ensured that the calculated XMCD spectrum of [Cu­(H_2_O)_6_]^2+^ is signed in agreement with experimental measurements of Cu­(II) compounds.

[Cu_2_(OAc)_4_(H_2_O)_2_]–Ferromagnetic and Antiferromagnetic coupling situations

We now build on top of the previous section and analyze how the magnetic coupling between two Cu­(II) centers affects the L_2,3_-edge absorption and XMCD spectra. For that we consider the model dimer [Cu_2_(OAc)_4_(H_2_O)_2_] (Figure 8a), which has been magnetically characterized as containing antiferromagnetically coupled Cu centers.^126,127^

**Figure 8:**
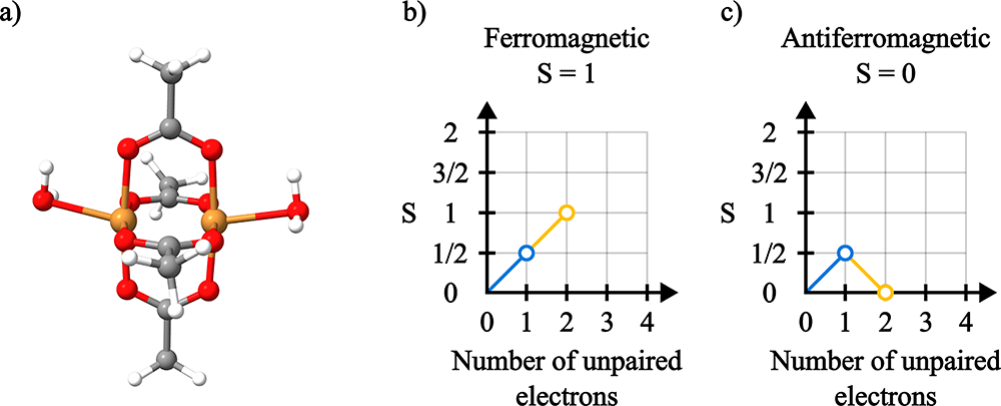
(a) Molecular geometry of [Cu_2_(OAc)_4_(H_2_O)_2_]. Branching diagram representations of the reference CSF for the (b) ferromagnetic and (c) antiferromagnetic coupling between the spins of the two Cu centers.

To probe the effects of different spin coupled ground states, GS-ROCIS calculations were performed for both *S* = 1 and *S* = 0 ground states, reflecting the scenarios of ferro- and antiferromagnetically coupled Cu centers, respectively. The ground state wave functions were obtained through CSF-ROHF calculations which were converged to the CSFs shown in Figure 8. The SOMOs in both scenarios are located in the $3d_{x^{2}-y^{2}}$ orbitals of the respective Cu centers (Figure S4).

For the GS-ROCIS calculation, the question arises on which orbitals should comprise the electron donor space, where it is possible to include both Cu centers into a single calculation, or perform individual calculations for each of the centers. In both spin coupling situations, the choice of Cu center set as the absorber species results in no difference in the obtained spectra reflecting the similar local chemical environment of both Cu atoms. The inclusion of both coppers into the donor space results in spectra intensities equivalent to the sum of the individual spectra (Figure S5).

The calculated L_2,3_-edge absorption spectra for both spin coupling situations are identical (Figure 9). Analysis of the excitations contributing to both the L_2_ and L_3_ edges shows that these are constituted by excitations from the Cu 2*p* orbitals into the $3d_{x^{2}-y^{2}}$ , which produce excited states in both singlet and triplet spin multiplicities (Table S3) that mix through the SOC operator. For both ferro and antiferromagnetic ground states, the excited states are the same.

**Figure 9:**
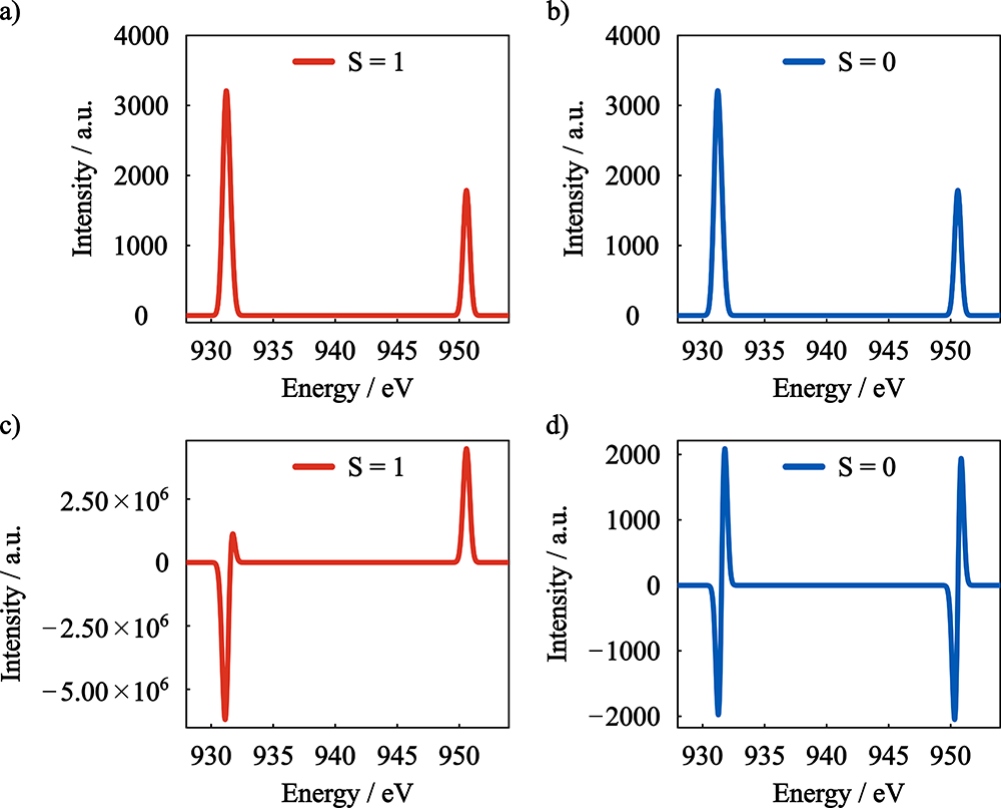
GS-ROCIS calculated L_2,3_-edge absorption for both (a) ferro- and (b) antiferromagnetic coupling situations of [Cu_2_(OAc)_4_(H_2_O)_2_]. GS-ROCIS calculated XMCD spectra (B = 3T and *T* = 4K) for (c) ferro- and (d) antiferromagnetic coupling situations. Basis set: x2c-TZVPall, Gaussian broadening: 0.62 eV.

Only by application of an external magnetic field differences arise as can be seen in the XMCD spectra in Figure 9c,d.

For the *S* = 1 case, the calculated XMCD spectra is similar to the one obtained for the [Cu­(H_2_O)_6_]^2+^ complex presented in the previous section. In contrast, for the *S* = 0 case both L_3_ and L_2_-edges have derivative shaped XMCD signals with intensities significantly lower than observed for the *S* = 1 system.

Since the analysis of the core excited states involved in the observed electronic transitions shows that both spin coupling situations have the same set of excited states, the observed differences in the calculated XMCD spectrum can be attributed to the different spin coupling situation of the ground state.

For the triplet ground state, the Zeeman effect causes the splitting of the three *M*_s_ components, which will have different Boltzmann populations depending on the temperature of the system. As a result, in lower temperatures, the electronic transitions from the lower Zeeman state will be more intense than of the less populated states, resulting in a stronger absorption of a given polarization of the incident radiation. Since a singlet ground state is not affected by the Zeeman effect, the absorption of circularly polarized radiation depends only on the nature of the excited states and it will not be affected by temperature.

We can probe the temperature dependency of the two spin coupling cases, by performing a series of GS-ROCIS calculations with varying temperature and fixed magnetic field. The obtained XMCD spectra are shown in Figure 10, where the intensity of the signals varies only for the *S* = 1 system, while it remains constant in the *S* = 0 system.

**Figure 10:**
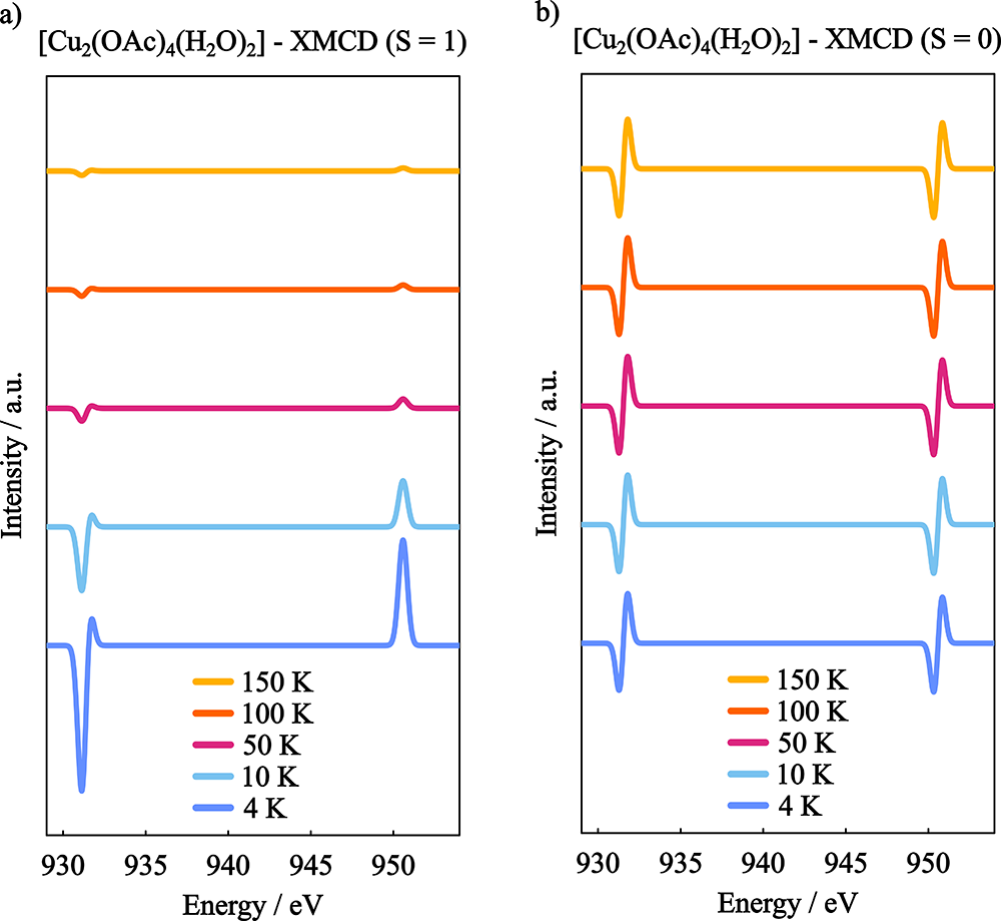
GS-ROCIS calculated XMCD spectra with varying temperature of the [Cu_2_(OAc)_4_(H_2_O)_2_] in (a) the *S* = 1 and (b) the *S* = 0 ground state situations with a constant field B = 3T.

In this example we demonstrated how the different spin coupling situation between metal centers can alter drastically the XMCD spectra, even when no discernible differences are observed in the L_2,3_-edge absorption spectra.

### L_2,3_-Edge XMCD of [(F_8_TPP)­Fe­(μ-O)­Cu­(TMPA)]^+^

Following the discussion of the [Cu_2_(OAc)_4_(H_2_O)_2_] model, which features antiferromagnetic coupling between two equivalent Cu­(II) centers, we now turn to a heterobimetallic dimer in which magnetic exchange occurs between two chemically and electronically distinct metal ions.

The dimer [(F_8_TPP)­Fe­(μ-O)­Cu­(TMPA)]^+^ (Figure 11a), where F_8_TPP = tetrakis­(2,6-difluorophenyl)­porphyrinate and TMPA = tris­(2-pyridylmethyl)­amine, consists of a Fe­(III) center with local $S=\frac{5}{2}$ and a Cu­(II) center with local $S=\frac{1}{2}$ that are experimentally characterized as antiferromagnetically coupled resulting in a *S* = 2 ground state.^118^ The measured Cu XMCD of this dimer shows opposite signs for the L_3_ and L_2_ (positive and negative respectively) absorption bands when compared to Cu monomers, where the L_3_ has a negative sign and the L_2_ a positive sign.^24,128^ This opposite behavior is attributed to the antiferromagnetic coupling between the Cu and Fe centers.^24^

**Figure 11:**
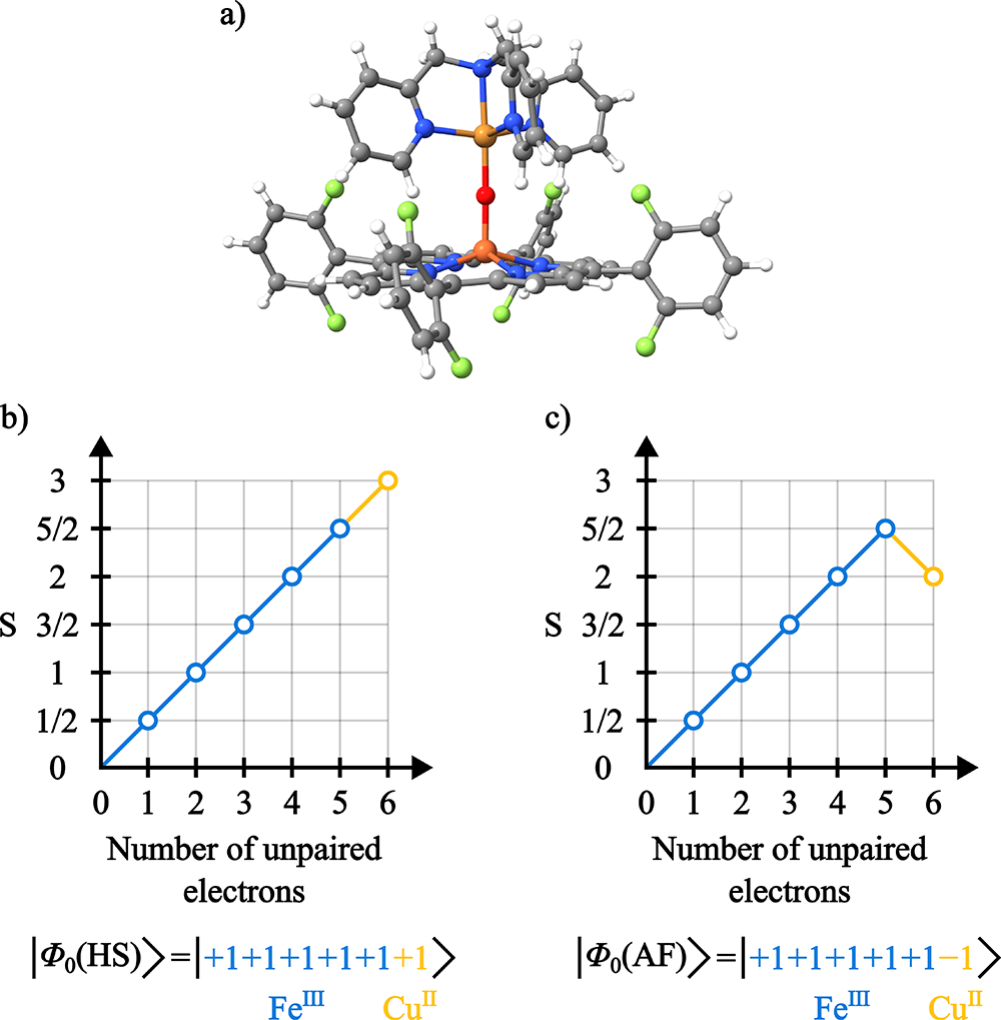
(a) Molecular geometry of the [(F_8_TPP)­Fe­(μ-O)­Cu­(TMPA)]^+^ complex. (b) Branching diagram representing the reference CSF for the ferromagnetic spin coupling between the metal centers, where HS stands for high-spin. (c) Branching diagram representing the reference CSF for the antiferromagnetic spin coupling between the metal centers.

To simulate the L_2,3_-edge absorption and XMCD spectra of the Cu and Fe centers under experimentally relevant conditions, GS-ROCIS calculations were performed using a magnetic field of 6T and a temperature of 2.2K^24^. Both ferro (*S* = 3) and antiferromagnetic (*S* = 2) coupling between the metal centers were considered in order to observe any spectral changes resulting from the different spin coupling situation. The ground state wave functions were obtained by converging CSF-ROHF calculations to the CSFs representative of the two magnetic coupling situations (Figure 11b,c).

For each ground state wave function two GS-ROCIS calculations were performed, where excitations from the core 2p orbitals of the Cu and Fe are probed separately. In all situations the acceptor orbital space comprises the entirety of the SOMOs and VMOs of the complex.

The calculated Cu XMCD spectra for both spin coupling situations are shown in Figure 12a together with the experimental spectrum from ref 128. It is readily seen that only the antiferromagnetically coupled calculation correctly predicts the signs of the L_3_ and L_2_ edges in the XMCD spectrum, although not with the correct intensity relation between the two edges.

**Figure 12:**
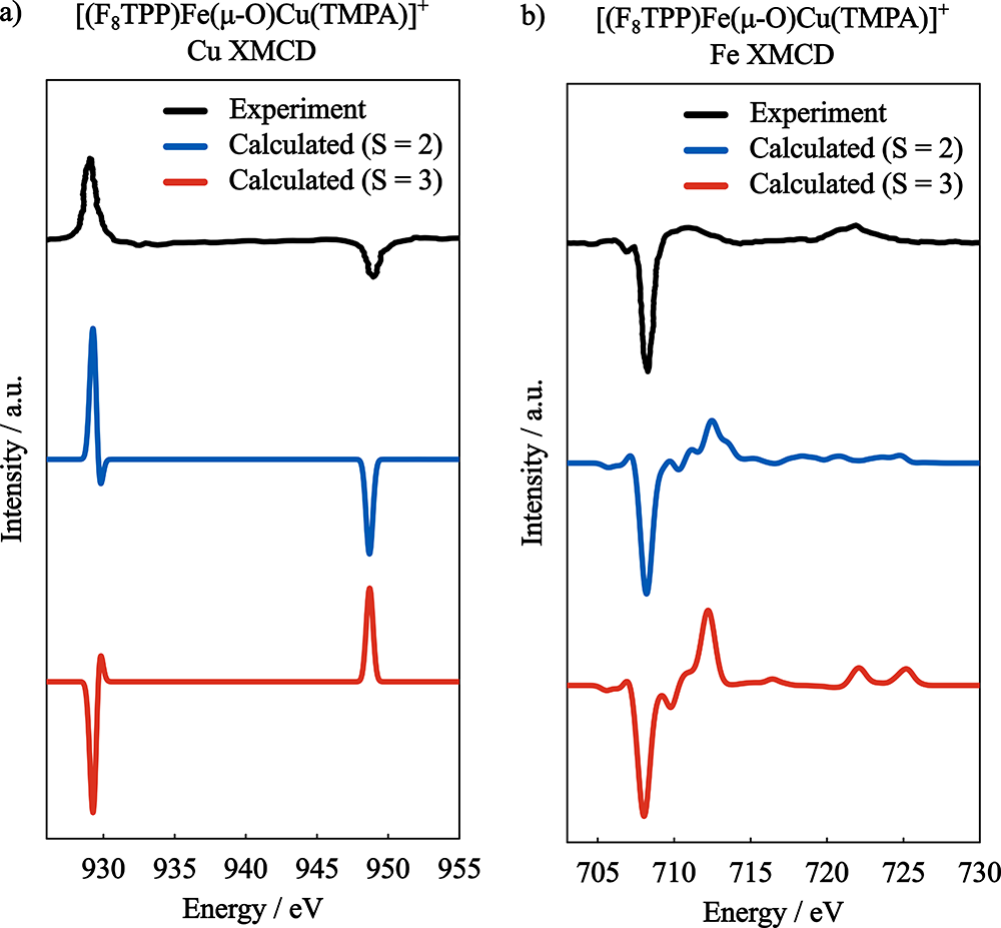
Experimental an GS-ROCIS calculated XMCD spectra of the (a) Cu center and (b) Fe center of [(F_8_TPP)­Fe­(μ-O)­Cu­(TMPA)]^+^. Basis set: x2c-TVPall, Gaussian broadening: 0.62 eV for Cu and 0.8 eV for Fe. Cu calculated spectra were shifted by −2.0 eV, Fe calculated spectra were shifted by −2.5 eV. Cu experimental spectrum was digitized from ref 128 and Fe experimental spectrum was digitized from ref 24

The L_2,3_-edge XAS and XMCD spectra of Fe­(III) are considerably more complex than the spectra of Cu­(II), due to the multiplets arising from the 2*p* ^5^3*d* ^6^ electronic configurations of the excited states, resulting in more features in the resulting spectra.

The Fe XMCD spectra are presented in Figure 12b, where it can be seen that the spin coupling situation does not have a drastic effect in the calculated spectra, both spin cases resulting in qualitatively correct spectra.

The similar Fe XMCD spectra for both spin coupling situations can be better understood by an analysis of the branching diagrams for both systems (Figure 11b,c). By definition, the ${Ê}_{q}^{p}$ operator allows for spin recouplings only inside the excitation range *p*, *q*. In the case of excitations from the Fe core 2p electrons into the Fe SOMOs, this restriction on spin recouplings leads to the same number of excited CSFs, all with the same spin coupling situation of the Fe SOMOs, which result in similar excited states in both systems. In contrast, excitations in the Cu center generate different numbers of excited CSFs, due to the large number of possible spin recouplings in the antiferromagnetic coupled system, which results in different calculated excited states.

Moreover, the transition densities between the nonrelativistic reference and core excited CSFs (Table 3) on the Fe center have the same value and sign for both spin coupling situations, while the transitions in the Cu center have opposite signs and different values between the *S* = 3 and *S* = 2 systems, which can be the source of the observed opposite signed Cu XMCD spectra and same sign Fe XMCD spectra.

**Table 3:** Transition Densities between the Non-Relativistic Reference and 2*p* to 3*d* Excited CSFs for Both Spin Coupling Situations of [(F_8_TPP)­Fe­(μ-O)­Cu­(TMPA)]^+^ ^a^

S = 3	S = 2
⟨Φ07|Êqp|Φ7Fe,2pFe,1−SOMO⟩=−1	⟨Φ05|Êqp|Φ5Fe,2pFe,1−SOMO⟩=−1
⟨Φ07|Êqp|Φ7Fe,2pFe,2−SOMO⟩=1	⟨Φ05|Êqp|Φ5Fe,2pFe,2−SOMO⟩=1
⟨Φ07|Êqp|Φ7Fe,2pFe,3−SOMO⟩=−1	⟨Φ05|Êqp|Φ5Fe,2pFe,3−SOMO⟩=−1
⟨Φ07|Êqp|Φ7Fe,2pFe,4−SOMO⟩=1	⟨Φ05|Êqp|Φ5Fe,2pFe,4−SOMO⟩=1
⟨Φ07|Êqp|Φ7Fe,2pFe,5−SOMO⟩=−1	⟨Φ05|Êqp|Φ5Fe,2pFe,5−SOMO⟩=−1
⟨Φ07|Êqp|Φ7Cu,2pCu,SOMO⟩=1	⟨Φ05|Êqp|Φ5Cu,2pCu,SOMO(+++++−)⟩=−0.200
	⟨Φ05|Êqp|Φ5Cu,2pCu,SOMO(++++−+)⟩=−0.245
	⟨Φ05|Êqp|Φ5Cu,2pCu,SOMO(+++−++)⟩=−0.316
	⟨Φ05|Êqp|Φ5Cu,2pCu,SOMO(++−+++)⟩=−0.447
	⟨Φ05|Êqp|Φ5Cu,2pCu,SOMO(+−++++)⟩=−0.775

a Φ_Fe,2p_^Fe,N–SOMO^ represent excited CSFs resulting from the excitation of an electron from the Fe 2*p* orbital to the Fe *N* ^*th*^ SOMO, while Φ_Cu,2p_^Cu,SOMO^ represent the excited CSFs resulting from the excitation of an electron from the Cu 2*p* orbital to the Cu SOMO. The resulting spin coupling is presented inside parentheses.

L_2,3_-edge XAS and XMCD of [Fe^II^(SPh)_4_]^2–^, [Fe^III^(SDur)_4_]^−^ and [L_2_Fe^II,III^_2_S_2_]^3–^

The results of the previous section might suggest that in cases of antiferromagnetic coupled metal centers, the metal with a smaller magnetic moment will have its XMCD spectra orientation reversed, while the center with higher magnetic moment retains the same sign. However, there are known cases^129,130^ where this reasoning does not hold.

Kowalska et al.^130^ reported the L_2,3_-edge XAS and XMCD spectra of a series of iron–sulfur complexes. Among the reported spectra was the mixed-valent dimer [L_2_Fe^II,III^_2_S_2_]^3–^ with L = bis­(benzimidazolato) (Figure 14a), which is characterized experimentally as having a Fe­(II) center with 3*d* ^6^ electronic configuration and local *S* = 2, and a Fe­(III) center with 3*d* ^5^ electronic configuration and local $S=\frac{5}{2}$ that are antiferromagnetically coupled, resulting in a total $S=\frac{1}{2}$ and effective coupling constant *J*_eff_ = −134 cm^–1^.^131^ The authors demonstrate that the XMCD spectrum of this dimer cannot be accounted for by the simple model of reversing the sign and adding the XMCD spectra of mononuclear Fe­(II) and Fe­(III) complexes.

**Figure 13:**
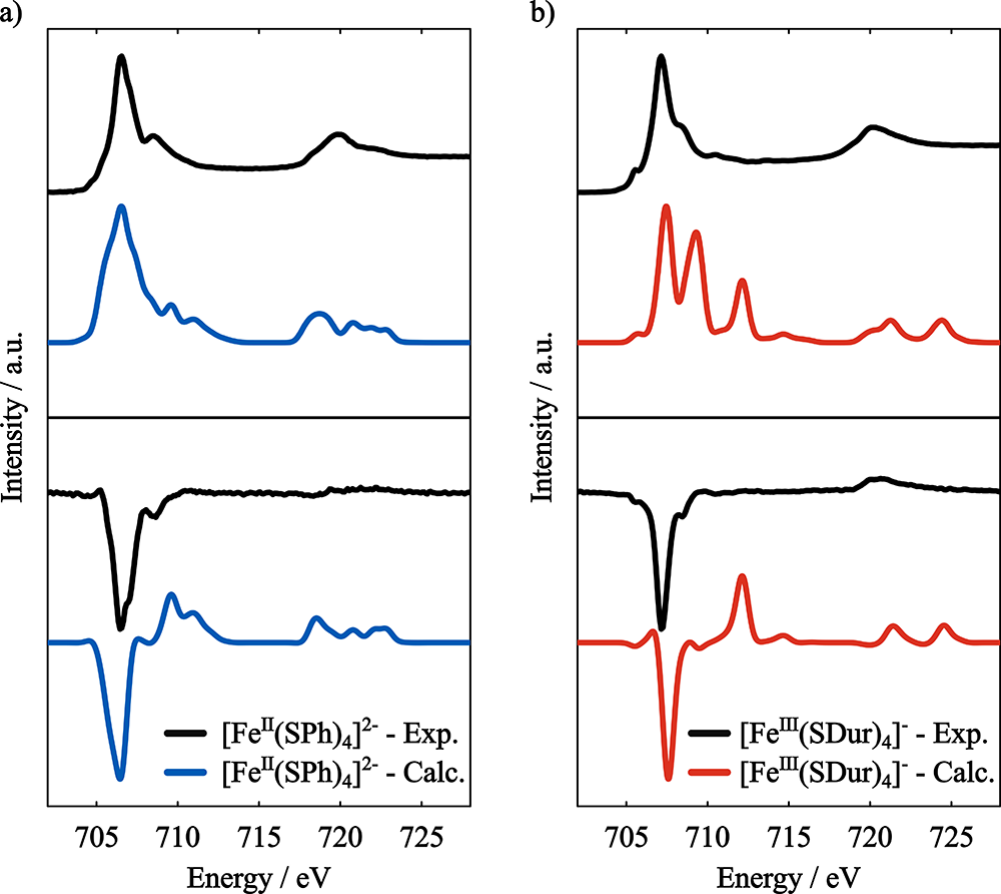
Experimental and calculated L_2,3_-edge XAS (top) and XMCD (bottom) spectra of a) [Fe^II^(SPh)_4_]^2–^ and b) [Fe^III^(SDur)_4_]^−^. Basis set: x2c-TZVPall, Gaussian broadening: 0.8 eV. Calculated spectra were shifted by −2.7 eV. Experimental data was extracted from ref 130.

**Figure 14:**
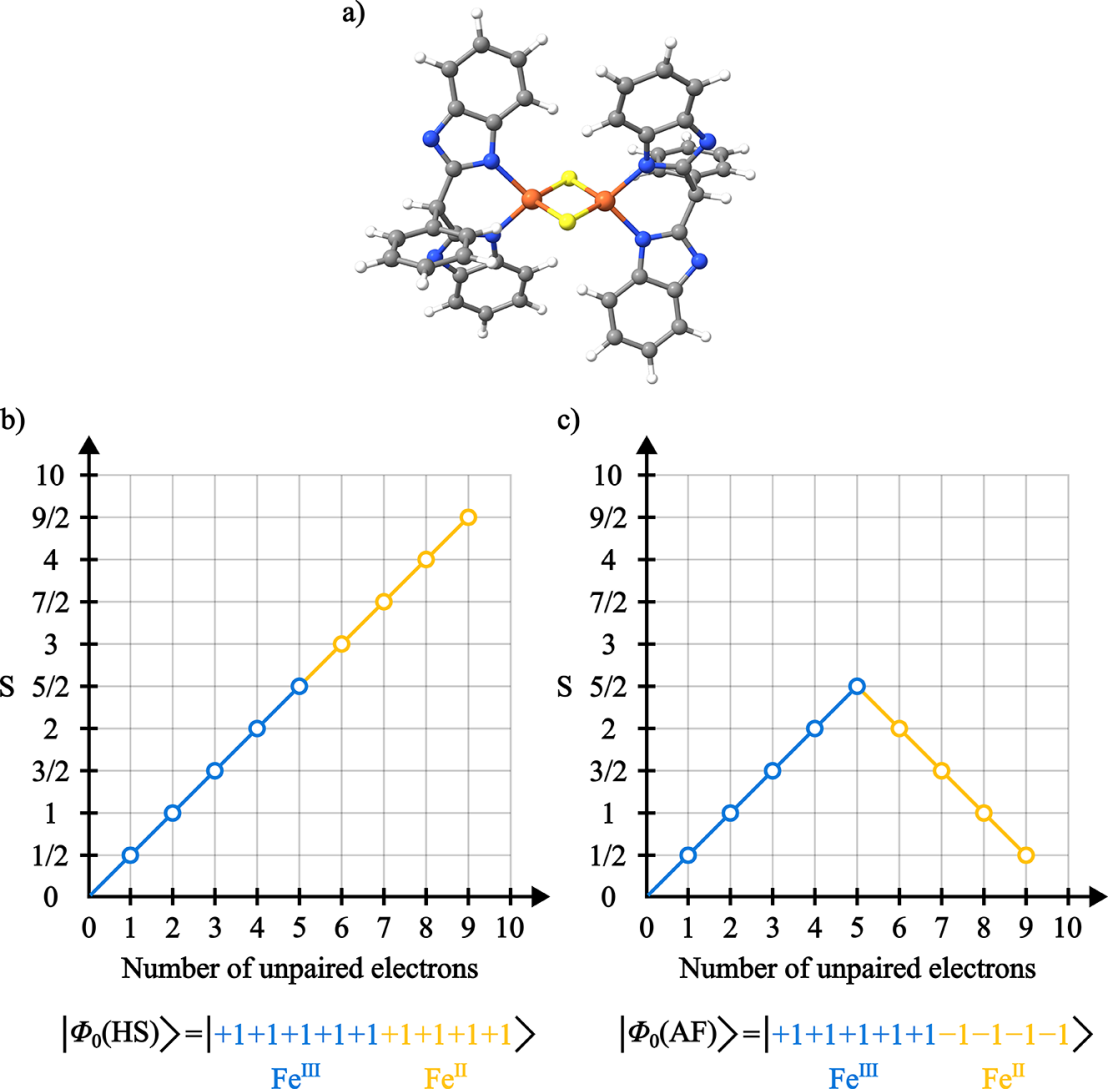
(a) Molecular structure and branching diagrams for the CSFs representing the (b) ferromagnetic and (c) antiferromagnetic coupling between the Fe centers in the [L_2_Fe^II,III^_2_S_2_]^3–^ dimer.

In this section we apply GS-ROCIS in further investigating the L_2,3_-edge XAS and XMCD spectra of the [L_2_Fe^II,III^_2_S_2_]^3–^ dimer.

We start by calculating the spectra of the mononuclear Fe complexes [Fe^II^(SPh)_4_]^2–^ and [Fe^III^(SDur)_4_]^−^ (SDur = 2,3,5,6-tetramethylbenzenethiolate) to evaluate the performance of GS-ROCIS for the two different oxidation states of the Fe center.

Experimentally, a shift of approximately 0.7 eV in the L_3_-edge energy was observed between the Fe­(II) and Fe­(III) complexes, accompanied by a reduced intensity of both the L_3_-and L_2_-edge absorption features in [Fe^III^(SDur)_4_]^−^, compared to [Fe^II^(SPh)_4_]^2–^. The XMCD spectra shows negative L_3_-edge features on both complexes, reflecting the higher absorption of LCP relative to RCP. The L_2_-edge is only observed in the XMCD spectra of [Fe^III^(SDur)_4_]^−^.

The calculated L_2,3_-edge absorption spectra of [Fe^II^(SPh)_4_]^2–^ (Figure 13a) shows good agreement with experiment, with correct relative intensities between the peaks and overall shape of the absorption band. The SOC splitting between the L_3_ and L_2_ is underestimated. The sign of the L_3_-edge XMCD feature is correctly predicted, while the predicted L_2_ feature is not observed in the experimental spectrum.

For the [Fe^III^(SDur)_4_]^−^ complex, the agreement between calculated and experimental L_2,3_-edge absorption spectra (Figure 13b) is not as good as it is for the Fe­(II) complex. The relative intensities between the peaks constituting the L_3_ feature are not correctly reproduced and are more spread in energy. Again, the SOC splitting is underestimated. The calculated difference between the L_3_-edge of the two complexes is ∼ 0.9 eV, in line with experimental results.

To evaluate the effect of the magnetic coupling between the iron centers on the final spectra, GS-ROCIS calculations were performed on both the $S=\frac{9}{2}$ and $S=\frac{1}{2}$ states, obtained by CSF-ROHF calculations converged to the CSFs represented by the branching diagrams shown in Figure 14. In both spin situations, GS-ROCIS calculations were performed individually on the Fe­(II) and Fe­(III) centers, with the final spectra being obtained as the sum of the individual spectra (Figures 15 and S8).

**Figure 15:**
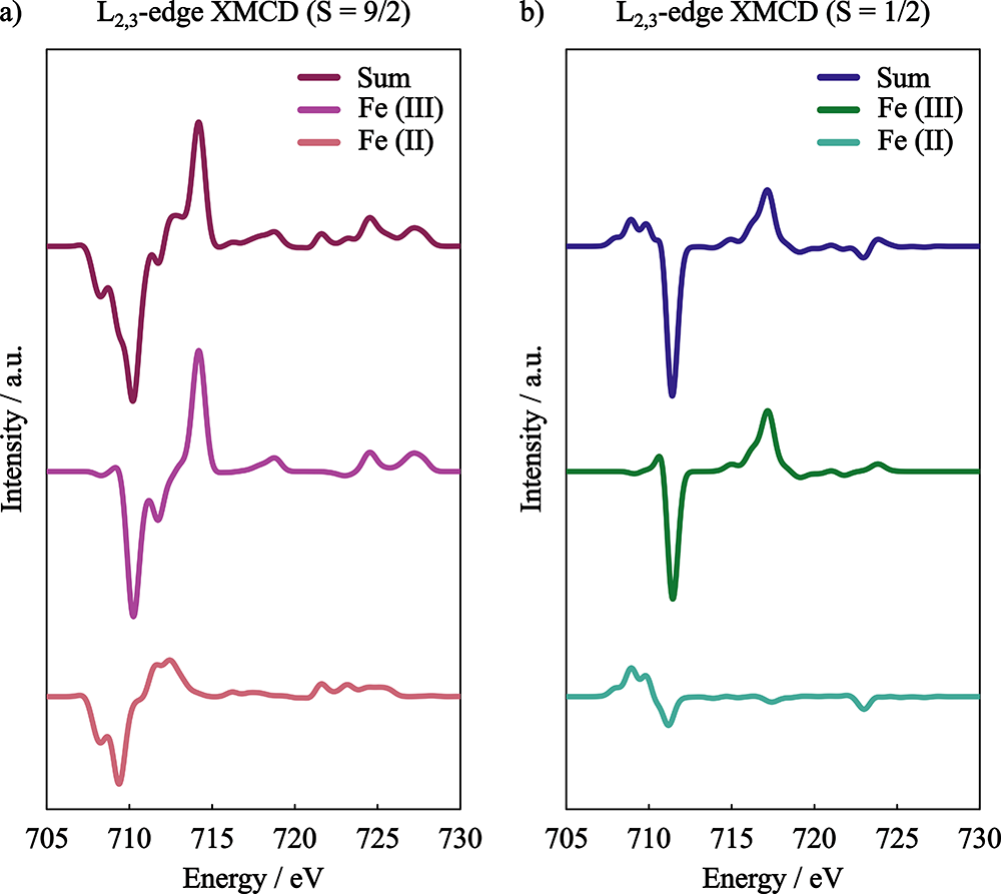
GS-ROCIS calculated L_2,3_-edge XMCD spectra of [L_2_Fe^II,III^_2_S_2_]^3–^ with (a) ferromagnetic and (b) antiferromagnetic coupling between the Fe centers. Basis set: x2c-TZVPall, Gaussian broadening: 0.8 eV.

From the individual contributions of the Fe­(II) centers to the XMCD spectra presented in Figure 15 we observe that there is a reversion of the sign of the features, accompanied by a drastic reduction in intensity in the antiferromagnetic coupling situation. This lower intensity causes the summed spectra to be dominated by the Fe­(III) spectrum. In the ferromagnetic case, the L_3_-edge XMCD features of both the Fe­(II) and Fe­(III) centers have the same sign, resulting in a summed spectra with a broader negative feature.

In contrast with what was observed in the previous sections, the calculated XMCD spectra of the Fe­(III), which is the majority spin center, differs between the two spin coupling situations, indicating that there is an interaction between the centers that is not present in the case of the [(F_8_TPP)­Fe­(μ-O)­Cu­(TMPA)]^+^ dimer, where the Fe­(III) center appears to not be sensitive to the Cu­(II) coupling situation. This interaction can be a result of covalency effects between the Fe centers and the bridging sulfur atoms, which are not properly captured by the ROHF reference wave function.

The summed L_2,3_-edge XAS and XMCD spectra of [L_2_Fe^II,III^_2_S_2_]^3–^ are shown in Figure 16 in comparison with experiment. Differently from the previous systems, the calculated L_2,3_ XAS spectra differ drastically between the two spin coupling situations.

**Figure 16:**
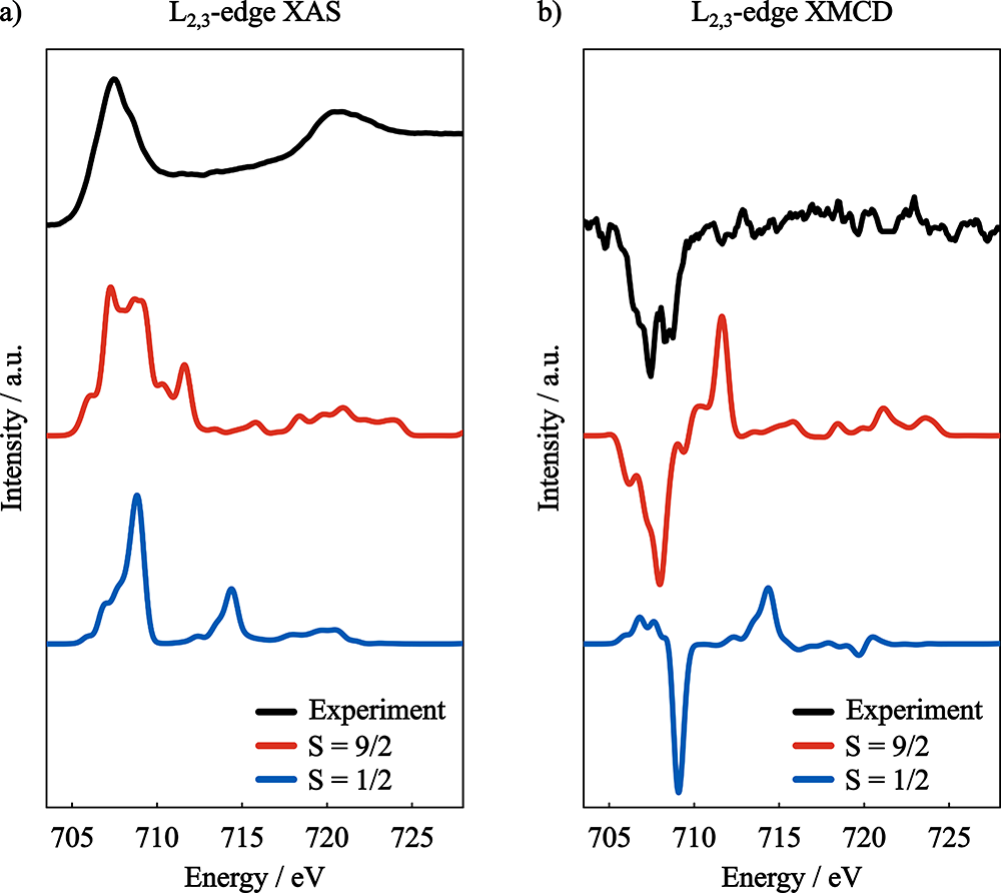
(a) Experimental (black) and GS-ROCIS calculated Fe L_2,3_-edge absorption spectra of [L_2_Fe_2_S_2_]^3–^ in ferromagnetic ( $S=\frac{9}{2}$ ) and antiferromagnetic ( $S=\frac{1}{2}$ ) spin coupling situations. (b) Experimental (black) and GS-ROCIS calculated XMCD spectra of the same complex in ferromagnetic and antiferromagnetic spin coupling situations. Basis set: x2c-TZVPall, Gaussian broadening: 0.8 eV. Shift: −2.8 eV. Experimental data was extracted from ref 130. Copyright 2017 American Chemical Society.

Interestingly, the calculated spectra of the ferromagnetic coupled system ( $S=\frac{9}{2}$ ) shows better agreement in both XAS and XMCD to the experimental result. However, as discussed previously, the reported magnetic susceptibility experiment^131^ show antiferromagnetic coupling between the Fe centers. This contrasting result may be the result of the lack of proper treatment of electron correlation within GS-ROCIS.

One attractive approach for treating this problem is the realization of GS-ROCIS in the framework of density functional theory (DFT). It has been shown that the high spin version of ROCIS benefits from its DFT version (ROCIS/DFT) in producing spectra with better experimental agreement.^67^ Efforts are currently underway in our group to tackle the shortcomings of GS-ROCIS in this regard.

## Conclusions

In this work we presented the inclusion of the spin–orbit coupling and Zeeman operators into the general spin ROCIS method in the framework of quasi-degenerate perturbation theory. The inclusion of the mentioned operators allows for computation of MCD and L-edge XAS and XMCD spectra of magnetically coupled transition metal systems. The performance of the method was investigated in chemical systems of varying spin coupling complexity.

The method was able to predict the MCD intensity sign of the isostructural complexes [LCr^III^(PyA)_3_Ni^II^]^2+^ and [LGa^III^(PyA)_3_Ni^II^]^2+^, corroborating with the idea that MCD can be used as an experimental means of identifying the minority spin species in a magnetically coupled dimer. Moreover, in situations of strong spin–orbit coupling, as the case of L_2,3_-edge X-ray spectroscopies, GS-ROCIS also performed satisfactory in predicting the XAS and XMCD spectra. For the coupled [Cu_2_(OAc)_4_(H_2_O)_2_] dimer, GS-ROCIS produced the expected behavior of the XMCD signals under temperature variation for both ferro- and antiferromagnetic coupling situations. GS-ROCIS also predicted the correct Cu XCMD signs of the L_3_ and L_2_ edges of the antiferromagnetically coupled [(F_8_TPP)­Fe­(μ-O)­Cu­(TMPA)]^+^ dimer.

We believe that the rigorous inclusion of the aforementioned operators into the GS-ROCIS method allows one to tackle spectroscopies of chemical systems of relevant size with high efficiency. Ongoing research in our group is directed toward enhancing the description of dynamic electron correlation within the method, with particular emphasis on spin-flip excitations and double-excitation character, which are critical for accurately modeling systems with complex spin-coupling phenomena. Further development in this direction is expected to significantly broaden the applicability and predictive power of GS-ROCIS in multiconfigurational transition-metal chemistry.

## Supplementary Material



## Data Availability

All output files of the calculations presented in this paper, as well as all the data necessary for reproduction of the calculated spectra are available at Edmond, the Open Research Data Repository of the Max Planck Society at the doi: 10.17617/3.3AQSEV

## Notes

Sample inputs, molecular geometries, molecular orbitals and calculated spectra indicated in the main text can be found in the Supporting Information (PDF)
