# Supplementary material for: General Spin Restricted Open-Shell Configuration Interaction Singles (GS-ROCIS): Implementation of Spin–Orbit Coupling and Zeeman Operators for Calculation of Optical and X‑ray Absorption and Magnetic Circular Dichroism Spectra of Magnetically Coupled Transition Metal Systems
Source: J Phys Chem A. 2025 Sep 30;129(40):9486–503. doi: 10.1021/acs.jpca.5c05086 (PMC12516740; doi:10.1021/acs.jpca.5c05086)
Supplement: Supplementary file 1 [file jp5c05086_si_001.pdf]

# Supporting Information

General Spin Restricted Open-Shell Configuration Interaction Singles (GS-ROCIS): Implementation of Spin–Orbit Coupling and Zeeman Operators for Calculation of Optical and X-Ray Absorption and Magnetic Circular Dichroism Spectra of Magnetically Coupled Transition Metal Systems

Tiago Leyser da Costa Gouveia[1], Lucas Lang[2], Dimitrios Maganas[1], Frank Neese[1*]

[1] Max-Planck-Institut für Kohlenforschung, Kaiser-Wilheim-Platz 1, 45470, Mülheim an der Ruhr, Germany

[2] Technische Universität Berlin, Institut für Chemie, Theoretische Chemie/Quantenchemie, Sekr. C7, Straße des 17. Juni 135, 10623, Berlin, Germany

Corresponding Author:

*neese@kofo.mpg.de

# Table of Contents


1 Sample inputs ........ S4

1.1 CSF-ROHF ........ S4

1.2 GS-ROCIS ........ S4

2 $[LCr^{III}(PyA)_3Zn^{II}]^{2+}$ ........ S6

2.1 Molecular Geometry – XYZ (Å) ........ S6

2.2 Experimental and GS-ROCIS calculated MCD spectrum ........ S7

3 $[LGa^{III}(PyA)_3Ni^{II}]^{2+}$ ........ S9

3.1 Molecular Geometry – XYZ (Å) ........ S9

3.2 Experimental and GS-ROCIS calculated MCD spectrum ........ S10

4 $[LCr^{III}(PyA)_3Ni^{II}]^{2+}$ ........ S12

4.1 Molecular Geometry – XYZ (Å) ........ S12

4.2 Experimental and GS-ROCIS calculated MCD spectrum ........ S13

5 $[Cu(H_2O)_6]^{2+}$ ........ S15

5.1 Molecular Geometry – XYZ (Å) ........ S15

5.2 Excited states obtained with GS-ROCIS ........ S15

6 $[Cu_2(OAc)_4(H_2O)_2]$ ........ S17

6.1 Molecular Geometry – XYZ (Å) ........ S17

6.2 Molecular orbitals obtained with CSF-ROHF ........ S17

6.3 Excited state obtained with GS-ROCIS ........ S18

6.4 GS-ROCIS Calculated $L_{2,3}$-edge XMCD ........ S19

7 $[(F_8TPP)Fe(\mu\text{-}O)Cu(TMPA)]^+$ ........ S20

7.1 Molecular Geometry – XYZ (Å) ........ S20

7.2 Molecular orbitals obtained from CSF-ROHF ........ S22

8 $[Fe^{II}(SPh)_4]^{2-}$ ........ S24

**8.1 Molecular Geometry – XYZ (Å)** ........ **S24**

**9 [$Fe^{III}(SDur)_4]^-$** ........ **S26**

**9.1 Molecular Geometry – XYZ (Å)** ........ **S26**

**10 [$L_2Fe^{II,III}{}_2S_2]^{3-}$** ........ **S29**

**10.1 Molecular Geometry – XYZ (Å)** ........ **S29**

**10.2 Molecular orbitals obtained from the CSF-ROHF calculation** ........ **S30**

**10.3 GS-ROCIS Calculated $L_{2,3}$-edge XAS** ........ **S31**

**11 References** ........ **S33**

# 1 Sample inputs

All calculation inputs can be found at Edmond, the Open Research Data Repository of the Max Planck Society at the doi: 10.17617/3.3AQSEV.

Here we give sample inputs that can be used as base for the calculations presented in this paper.

## 1.1 CSF-ROHF

```
!x2c x2c-TZVPall TightSCF MOREAD

%moinp "cu2aco_hsrohf.loc" #read the localized MOs from the HS-ROHF calculation

%scf
  HFTyp ROHF
  ROHF_CASE AF_CSF
  ROHF_AFORBS 1,1
end
```

## 1.2 GS-ROCIS

```
!x2c x2c-TZVPall AutoAux MOREAD NoIter

%moinp "cu2aco_afrohf.gbw" #read the CSF-ROHF orbitals for the AF case

%scf
  HFTyp ROHF
  ROHF_CASE AF_CSF
  ROHF_AFORBS 1,1
end

%rocis
  DoGenROCIS true
  ReferenceMult 1
  NRoots 100
  OrbWin 4,6,0,2000
  DoLoc true
  LocOrbWin 4,9
  DoPNO true
  XASelems 0
  TCutPNO 1e-13
  Rel
    DoSOC true
    DoQDPT true
    DoMCD true
```

```
    B 30000
    Temperature 4
  end
  DoRI true
  DoHigherMult true
  DoLowerMult false
  DoDipoleVelocity true
  DoFullSemiClassical true
  DecomposeFosc true
end
```

# 2 $[LCr^{III}(PyA)_3Zn^{II}]^{2+}$

## 2.1 Molecular Geometry – XYZ (Å)

| | | | |
|---|---|---|---|
| Zn | -1.82454288108850 | 0.99242041048529 | -0.88687601061968 |
| Cr | 1.65745564222268 | 1.84011873825004 | -0.27083428190994 |
| N | -2.91436781512610 | -0.44372961956929 | 0.34291213274661 |
| N | -3.41035255662712 | 2.47428524702072 | -0.82632143554357 |
| N | -0.80065653384811 | 2.90091630458420 | -1.17308745150189 |
| N | -0.01761321231064 | 0.11912351945003 | -1.76066476818758 |
| N | -2.49420765721042 | 0.10860180836508 | -2.75019486077095 |
| N | -0.70295188592747 | 0.90542533089010 | 0.98082478235049 |
| C | -0.23063017591826 | -0.69433262720418 | -2.74508801652358 |
| C | -1.57185793300390 | -0.71489629297151 | -3.31458490831950 |
| C | -3.71192879532680 | 0.19234837428297 | -3.30460433820712 |
| C | -4.08949127215190 | -0.55233511380585 | -4.42152963579822 |
| C | -3.15678230077747 | -1.42416895421667 | -4.98580337958625 |
| C | -1.88176375338091 | -1.50313037234818 | -4.43061611887602 |
| C | -3.96187094056867 | -1.20193172096145 | -0.00886339927381 |
| C | -4.67554410562683 | -1.98123171118808 | 0.90176330933734 |
| C | -4.28057282355160 | -1.96777264455212 | 2.24053093599745 |
| C | -3.18201637449013 | -1.19733953430747 | 2.61439795024414 |
| C | -2.50823130105052 | -0.44985852458375 | 1.63973942316535 |
| C | -1.30446125774919 | 0.30637751088838 | 1.95833577968402 |
| H | -4.40818652279334 | 0.88877444196953 | -2.83460228821451 |
| H | -5.09198150529877 | -0.44607377814094 | -4.83392882723547 |
| H | -3.41739834214340 | -2.02955009120383 | -5.85456724396881 |
| H | -1.12251831273468 | -2.15904684256366 | -4.85741050377209 |
| H | -4.23517258507568 | -1.18637235645386 | -1.06525896685613 |
| H | -5.51909364901379 | -2.58158154236036 | 0.56381947409634 |
| H | -4.81805890343260 | -2.55777399485778 | 2.98338944454675 |
| H | -2.83521084189167 | -1.17641295614246 | 3.64787333944522 |
| C | -4.70648034514192 | 2.27748573854424 | -0.54646713354890 |
| C | -5.67130812361383 | 3.27632609990180 | -0.67373720897515 |
| C | -5.26768919171416 | 4.53741168974506 | -1.11701120866510 |
| C | -3.00755806932176 | 3.70832804316195 | -1.22873646562719 |
| C | -1.57753096580241 | 3.90591777423907 | -1.42411033408057 |
| C | -3.92056616400056 | 4.75806202100547 | -1.39340018937830 |
| H | -3.56765804648134 | 5.73497652591491 | -1.72475560589170 |
| H | -5.99342285366976 | 5.34209062311798 | -1.23732561343009 |
| H | -4.97738479062803 | 1.27802318620181 | -0.20249553215720 |
| H | -6.71185606205594 | 3.06477965481973 | -0.43099349486708 |
| O | 0.44644110882072 | 1.53196765748036 | 1.22932030147342 |
| O | 1.21871004679370 | 0.22474152070069 | -1.27562896531424 |
| O | 0.51721016993446 | 3.07725477340056 | -1.26317037228535 |
| N | 2.51384505265154 | 3.52583694786111 | 0.76109630441502 |
| N | 3.18479732629823 | 0.76394701272317 | 0.80158167642258 |

| | | | |
|---|---|---|---|
| N | 3.28125821317352 | 2.21071392928870 | -1.63726706888787 |
| C | 3.54646582568124 | 3.03660865069446 | 1.73770316186152 |
| C | 3.34928024278628 | 1.56004086262407 | 2.05382012756947 |
| H | 4.19536141913010 | 1.18153531055185 | 2.65287907273245 |
| H | 2.42896585590988 | 1.41609103594668 | 2.63460164134482 |
| H | 3.49059367863444 | 3.62219911953773 | 2.66668689944247 |
| H | 4.54446184444691 | 3.21993522455043 | 1.32082020681195 |
| C | 4.14507740824907 | 1.00259820427224 | -1.48636544802203 |
| C | 4.44332262758745 | 0.73302815741719 | -0.01819319720049 |
| H | 5.08470742927452 | 1.11688562638679 | -2.05379054491673 |
| H | 3.58501004810068 | 0.16409547521865 | -1.92094154790741 |
| H | 5.14544443737737 | 1.47471324525240 | 0.38179885338499 |
| H | 4.93645928247482 | -0.24408817208580 | 0.08711940844936 |
| C | 3.11830152461155 | 4.33480878712956 | -0.33909057002508 |
| C | 3.99680094139936 | 3.46790873906255 | -1.23001200833358 |
| H | 4.29381626305755 | 4.03645107177722 | -2.12313357692097 |
| H | 4.92795454472933 | 3.19623963828648 | -0.71769903937787 |
| H | 3.69959197223421 | 5.18012378228268 | 0.06741759490901 |
| H | 2.28078655622435 | 4.74406133060553 | -0.91882894866302 |
| C | 2.80520533961677 | 2.31731234957276 | -3.04025988280140 |
| H | 2.23823314901823 | 1.41510585698896 | -3.29377262881835 |
| H | 3.65914489916896 | 2.42122270147693 | -3.72849359784185 |
| H | 2.14504601658284 | 3.18607890637205 | -3.13392961196610 |
| C | 2.75435060677759 | -0.61763129001883 | 1.13555742676374 |
| H | 1.80334517206232 | -0.57480826619753 | 1.67691250209929 |
| H | 3.51237329699185 | -1.11608948207828 | 1.76053026417452 |
| H | 2.60657586078692 | -1.18393459018612 | 0.20991849284775 |
| C | 1.47673892804910 | 4.32458384221059 | 1.46270486265709 |
| H | 1.00543291178833 | 3.70608603119279 | 2.23381795788593 |
| H | 0.71330291583985 | 4.62908098590689 | 0.73892075262793 |
| H | 1.92506687703788 | 5.21923774496142 | 1.92366188468133 |
| H | 0.56984124620469 | -1.30331704527252 | -3.17691206102243 |
| H | -0.90049927171479 | 0.31341606095498 | 2.97537092478770 |
| H | -1.18139855946624 | 4.88270389774335 | -1.71745859686475 |

## 2.2 Experimental and GS-ROCIS calculated MCD spectrum

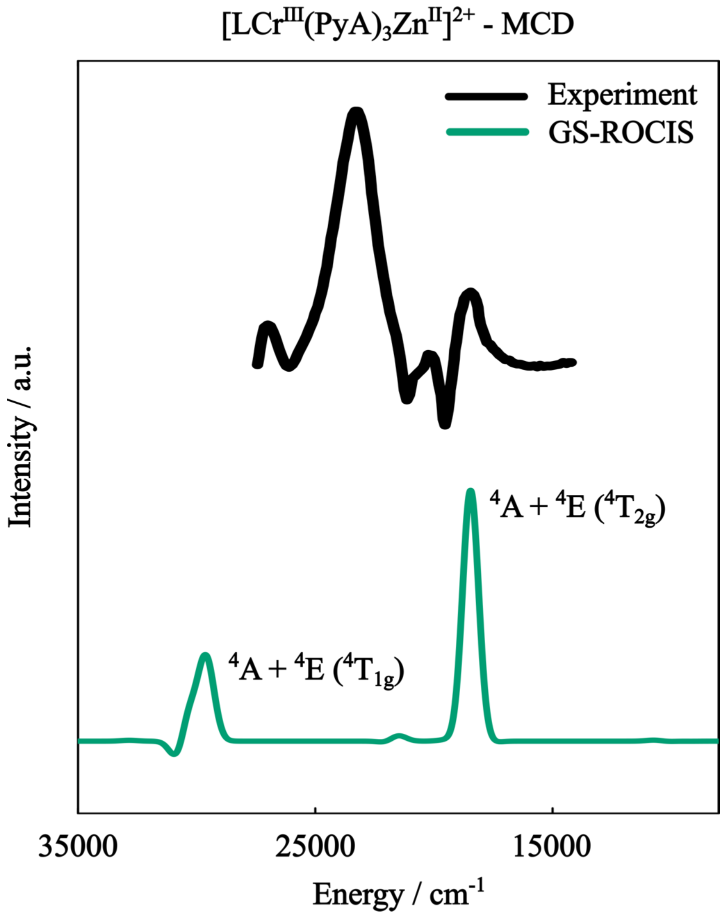


Figure S1: Experimental and GS-ROCIS calculated MCD spectrum of $[LCr^{III}(PyA)_3Zn^{II}]^{2+}$. Experimental data was digitized from reference 1 Calculated spectrum was shifted by −300 $cm^{-1}$ and a Gaussian broadening of 800 cm-1 was used.

# 3 $[LGa^{III}(PyA)_3Ni^{II}]^{2+}$

## 3.1 Molecular Geometry – XYZ (Å)

| | | | |
|---|---|---|---|
| Ni | -1.71315443376540 | 1.01067982636531 | -0.85848983983793 |
| Ga | 1.60026112533122 | 1.81678320351076 | -0.27475385111385 |
| N | -2.75075819579180 | -0.50000728143691 | 0.18435606532123 |
| N | -3.30655727446980 | 2.37259105174277 | -0.63067677625186 |
| N | -0.80938817308858 | 2.78610349442131 | -1.31076730592321 |
| N | -0.08562969414281 | 0.03438708063255 | -1.61509942891891 |
| N | -2.44441101574341 | 0.33945212376739 | -2.71640827889086 |
| N | -0.78813740569864 | 1.05269224669197 | 0.96311803406752 |
| C | -0.28521319272369 | -0.67092709652364 | -2.68435140181085 |
| C | -1.59475530410317 | -0.55153681183660 | -3.29976151073162 |
| C | -3.63991758772541 | 0.55281272036600 | -3.28396438945998 |
| C | -4.05843120094352 | -0.10744639618561 | -4.43946433250041 |
| C | -3.19866027335727 | -1.03499586373026 | -5.03024029952128 |
| C | -1.94963783015979 | -1.25831357062835 | -4.45513152701664 |
| C | -3.71826668427606 | -1.31955779083114 | -0.25060428994727 |
| C | -4.44958844095368 | -2.14770357544269 | 0.60124582487239 |
| C | -4.16674449100565 | -2.12118823552058 | 1.96799931201905 |
| C | -3.15661346356201 | -1.28094199243640 | 2.43094644250388 |
| C | -2.45905796187482 | -0.48416930969689 | 1.51502073904822 |
| C | -1.34428851947194 | 0.35944572518599 | 1.90670614939419 |
| H | -4.28496868226509 | 1.28057897975189 | -2.79078617993631 |
| H | -5.03865385849875 | 0.10809289255982 | -4.86270876644459 |
| H | -3.49563706534064 | -1.57332952160604 | -5.93048845385030 |
| H | -1.24736081049376 | -1.96787478893773 | -4.89316840986535 |
| H | -3.91286377089211 | -1.30999456441990 | -1.32349890172173 |
| H | -5.22184094418908 | -2.79868178965025 | 0.19348288502362 |
| H | -4.72237893498690 | -2.75084866303700 | 2.66315227214369 |
| H | -2.89991967144361 | -1.23941476723029 | 3.48966440468840 |
| C | -4.56533902816892 | 2.14461250404993 | -0.23021943680310 |
| C | -5.56852182783796 | 3.11163910772832 | -0.29847567994748 |
| C | -5.25186620307163 | 4.37142487985033 | -0.80981117919953 |
| C | -2.98879822225702 | 3.60568433295122 | -1.11452662503260 |
| C | -1.59123653391621 | 3.81070411016579 | -1.44999510445438 |
| C | -3.94484470224204 | 4.62315004054228 | -1.22076164297838 |
| H | -3.65719489969031 | 5.59711625590630 | -1.61722930212856 |
| H | -6.01234022424071 | 5.14911506381349 | -0.88266411065739 |
| H | -4.77486768160938 | 1.14779871986220 | 0.15890802177113 |
| H | -6.57446545372378 | 2.87526525906745 | 0.04599310319388 |
| O | 0.32933351268050 | 1.73541574880717 | 1.24243287915120 |
| O | 1.14616712940732 | 0.06868181030694 | -1.09107486644644 |
| O | 0.50907699512357 | 2.96031190810227 | -1.47041178206159 |
| N | 2.47749205596882 | 3.60382705455928 | 0.57931620944429 |
| N | 3.09041307723758 | 0.83750858745601 | 0.95929536924774 |
| N | 3.26440943827707 | 1.99288139453036 | -1.64977595607262 |

| | | | |
|---|---|---|---|
| C | 3.47744122518526 | 3.20810309900176 | 1.62186332428744 |
| C | 3.25097874311292 | 1.77704629032951 | 2.10434282744927 |
| H | 4.08725827034969 | 1.46601703934271 | 2.75229918942291 |
| H | 2.32405964184355 | 1.71645770593214 | 2.68951753153724 |
| H | 3.41494073803084 | 3.89283380110588 | 2.47933817464453 |
| H | 4.48739274423446 | 3.32543296272481 | 1.20963721825828 |
| C | 4.09272335061438 | 0.79552278974178 | -1.33084090453432 |
| C | 4.35359798895093 | 0.68707035136523 | 0.16944603355342 |
| H | 5.05107909359261 | 0.82580346059938 | -1.87522015699995 |
| H | 3.52653480804169 | -0.07716119900446 | -1.68151324606904 |
| H | 5.06932493465249 | 1.45241083740670 | 0.49384684353001 |
| H | 4.82071786404384 | -0.28347597654195 | 0.38846662261526 |
| C | 3.11917686613749 | 4.25782915248151 | -0.59620783974336 |
| C | 3.98972139605389 | 3.27415744475737 | -1.37468418723418 |
| H | 4.30140881807598 | 3.73790117997065 | -2.32115777282708 |
| H | 4.91235995227146 | 3.05098971142988 | -0.82484487916368 |
| H | 3.72569499876188 | 5.12231005081007 | -0.27926267895646 |
| H | 2.30332167460348 | 4.62657812683425 | -1.23135128000969 |
| C | 2.80967077223851 | 1.93690559267563 | -3.06430653194299 |
| H | 2.21409221850649 | 1.02960194462156 | -3.20874784465926 |
| H | 3.67536430490321 | 1.92359227798223 | -3.74453411442612 |
| H | 2.17929227325757 | 2.80576681457699 | -3.27815062418588 |
| C | 2.61247373879330 | -0.48399072067614 | 1.44532255999935 |
| H | 1.64703897890620 | -0.35166260571326 | 1.94477068637061 |
| H | 3.33739901236819 | -0.91670521352913 | 2.15221979545757 |
| H | 2.47609622238808 | -1.15617969425587 | 0.59257429415899 |
| C | 1.44189688050149 | 4.50162996350912 | 1.15603138078833 |
| H | 0.96240015782222 | 4.00735575760840 | 2.00679116668477 |
| H | 0.68181140742094 | 4.70165195148349 | 0.39371791604667 |
| H | 1.89744421657161 | 5.45001254472735 | 1.48057466321546 |
| H | 0.51347213081346 | -1.26441368389831 | -3.13734071174432 |
| H | -0.94728179579132 | 0.35526031378909 | 2.92541761171312 |
| H | -1.20455047003591 | 4.79281770053339 | -1.73507500333810 |

### 3.2 Experimental and GS-ROCIS calculated MCD spectrum

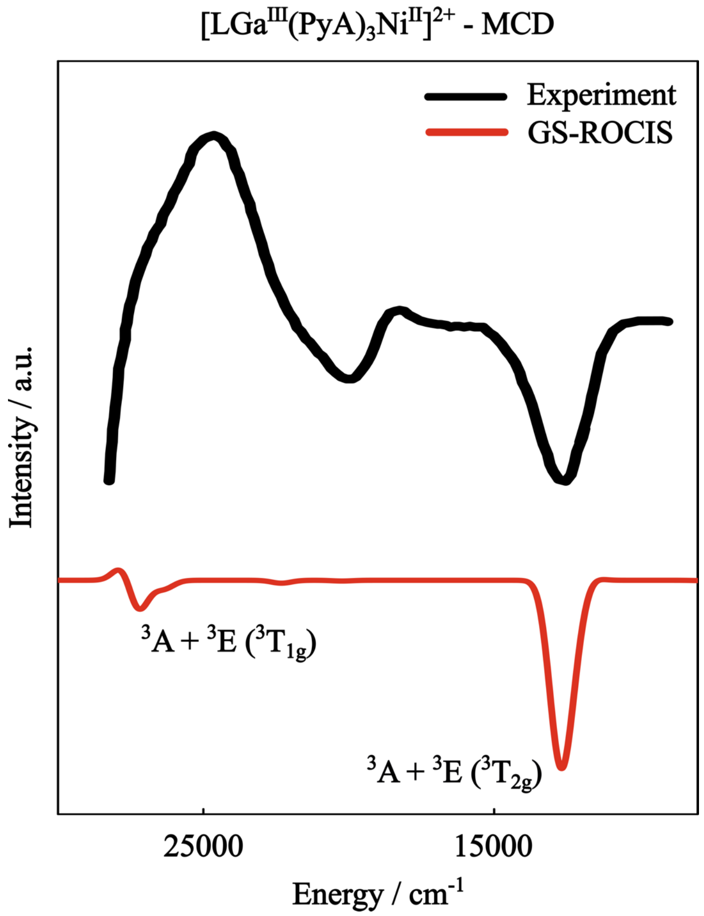


Figure S2: Experimental and GS-ROCIS calculated MCD spectrum of $[LGa^{III}(PyA)_3Ni^{II}]^{2+}$. Experimental data was digitized from reference 1. Calculated spectrum was shifted by +500 $cm^{-1}$ and a Gaussian broadening of 800 $cm^{-1}$ was used.

# 4 $[LCr^{III}(PyA)_3Ni^{II}]^{2+}$

## 4.1 Molecular Geometry – XYZ (Å)

| | | | |
|---|---|---|---|
| Ni | -1.70540871553713 | 1.01295339492939 | -0.86548319870910 |
| Cr | 1.62453965772200 | 1.82595665881819 | -0.26904155851857 |
| N | -2.74554229431221 | -0.49969633279964 | 0.16991279065325 |
| N | -3.30209890580217 | 2.36564557821694 | -0.63734816465963 |
| N | -0.80162856543037 | 2.79740175123213 | -1.28497596375320 |
| N | -0.06942843994728 | 0.05396694672548 | -1.63184303907597 |
| N | -2.43289373938852 | 0.33817164524097 | -2.71958955124052 |
| N | -0.77363455450464 | 1.03303702244454 | 0.95364113381698 |
| C | -0.28027006551509 | -0.68661120443504 | -2.67721487757221 |
| C | -1.59134009311255 | -0.57109921502733 | -3.28794929915453 |
| C | -3.62685544517087 | 0.55359423196696 | -3.28961622878912 |
| C | -4.05400243586791 | -0.12396510126565 | -4.43168903460394 |
| C | -3.20504104504909 | -1.07269793867751 | -5.00471630235603 |
| C | -1.95726226343289 | -1.29722725116600 | -4.42813492635523 |
| C | -3.71030977675743 | -1.31903822874865 | -0.27100325424769 |
| C | -4.45478723752233 | -2.13876821679346 | 0.57764836422393 |
| C | -4.18865585175075 | -2.10259288969214 | 1.94779925220799 |
| C | -3.18099398631638 | -1.26345873369038 | 2.41714624974598 |
| C | -2.46772571073753 | -0.47693521830902 | 1.50408812734902 |
| C | -1.35178197842522 | 0.36060952156351 | 1.90175457503192 |
| H | -4.26428737931370 | 1.29550454404268 | -2.80781983392255 |
| H | -5.03242106877370 | 0.09457547764887 | -4.85806346888174 |
| H | -3.51002161239935 | -1.62808219853674 | -5.89211651662041 |
| H | -1.26383894517782 | -2.02246690360701 | -4.85403532871135 |
| H | -3.89352023185846 | -1.31582914022523 | -1.34589237186731 |
| H | -5.22485824419033 | -2.78962483281816 | 0.16530217580758 |
| H | -4.75617414809870 | -2.72393644712766 | 2.64082266898343 |
| H | -2.93714268144024 | -1.21591447711655 | 3.47872707950355 |
| C | -4.56115523225028 | 2.13035601221735 | -0.24215506588221 |
| C | -5.56828903592534 | 3.09345251484150 | -0.30858346948602 |
| C | -5.25534908570556 | 4.35689794972387 | -0.81318625894668 |
| C | -2.98724826704260 | 3.60338600304640 | -1.11326444744965 |
| C | -1.59001973697313 | 3.81708953265458 | -1.44005270338396 |
| C | -3.94818419711799 | 4.61663712486561 | -1.21839021698947 |
| H | -3.66430621938276 | 5.59387032433890 | -1.60909997924501 |
| H | -6.01895308638982 | 5.13163645318266 | -0.88494992805158 |
| H | -4.76859250357485 | 1.13096243573858 | 0.14079110729727 |
| H | -6.57464294860421 | 2.85063755130198 | 0.03048374006383 |
| O | 0.34767887661205 | 1.69725671813057 | 1.21949318880838 |
| O | 1.15526326356131 | 0.11542945280157 | -1.11603802475050 |
| O | 0.51122851493340 | 2.96591907733459 | -1.41931682678591 |
| N | 2.47858700110848 | 3.59242298386646 | 0.60080829364415 |
| N | 3.09929447228630 | 0.84510143190886 | 0.94437241395805 |
| N | 3.27207430988151 | 2.02450548523058 | -1.62986599839204 |

| | | | |
|---|---|---|---|
| C | 3.48137957854763 | 3.19475867827700 | 1.64810563724219 |
| C | 3.25123999038659 | 1.76293839217952 | 2.11197580129626 |
| H | 4.07781210546680 | 1.43601788330369 | 2.76610333565969 |
| H | 2.31689749940495 | 1.69291108993030 | 2.68432194098326 |
| H | 3.41503969120079 | 3.87632220429145 | 2.50823425471610 |
| H | 4.49151721341576 | 3.31775336422711 | 1.23818609466522 |
| C | 4.11017181537183 | 0.82431164873460 | -1.33335942933701 |
| C | 4.37454134753303 | 0.70936964245240 | 0.16097962360705 |
| H | 5.06260968267865 | 0.86104118772007 | -1.88946224632275 |
| H | 3.54221086387697 | -0.04589570424186 | -1.68788773433832 |
| H | 5.07862131306238 | 1.48202183826709 | 0.49277754442333 |
| H | 4.85124298871831 | -0.25661580069223 | 0.38133946581723 |
| C | 3.12012494992768 | 4.27501973415066 | -0.56230006524858 |
| C | 3.99966492872744 | 3.30858277507983 | -1.34337890726250 |
| H | 4.31553139058134 | 3.77781322463951 | -2.28615400246579 |
| H | 4.92026009659974 | 3.08140196497904 | -0.79189302091926 |
| H | 3.71018739957492 | 5.14617962971598 | -0.22995172132854 |
| H | 2.30207495304385 | 4.64134217236997 | -1.19637471184478 |
| C | 2.82141573282313 | 1.99018818536459 | -3.04503901372016 |
| H | 2.24412017126431 | 1.07460747320411 | -3.21177815679531 |
| H | 3.68760811383391 | 2.00914543654128 | -3.72534079967312 |
| H | 2.17568199108664 | 2.85270431837951 | -3.24112177832907 |
| C | 2.63344720281770 | -0.48884863543636 | 1.40394090223364 |
| H | 1.66917718189895 | -0.37574747039930 | 1.91080807261011 |
| H | 3.36478718631726 | -0.93331818282179 | 2.09770200858794 |
| H | 2.50006204009022 | -1.14327130128380 | 0.53623810736376 |
| C | 1.44042973547682 | 4.47687524595390 | 1.19048025877442 |
| H | 0.95524785334469 | 3.96340019822976 | 2.02737031448497 |
| H | 0.68604394907678 | 4.69653724958982 | 0.42767860916987 |
| H | 1.89328077229372 | 5.41752416589762 | 1.54178376906259 |
| H | 0.51055002945551 | -1.30044465751678 | -3.11641400871068 |
| H | -0.96964804578611 | 0.36754582973177 | 2.92592238751258 |
| H | -1.20737208941793 | 4.79979472520372 | -1.72783785460735 |

## 4.2 Experimental and GS-ROCIS calculated MCD spectrum

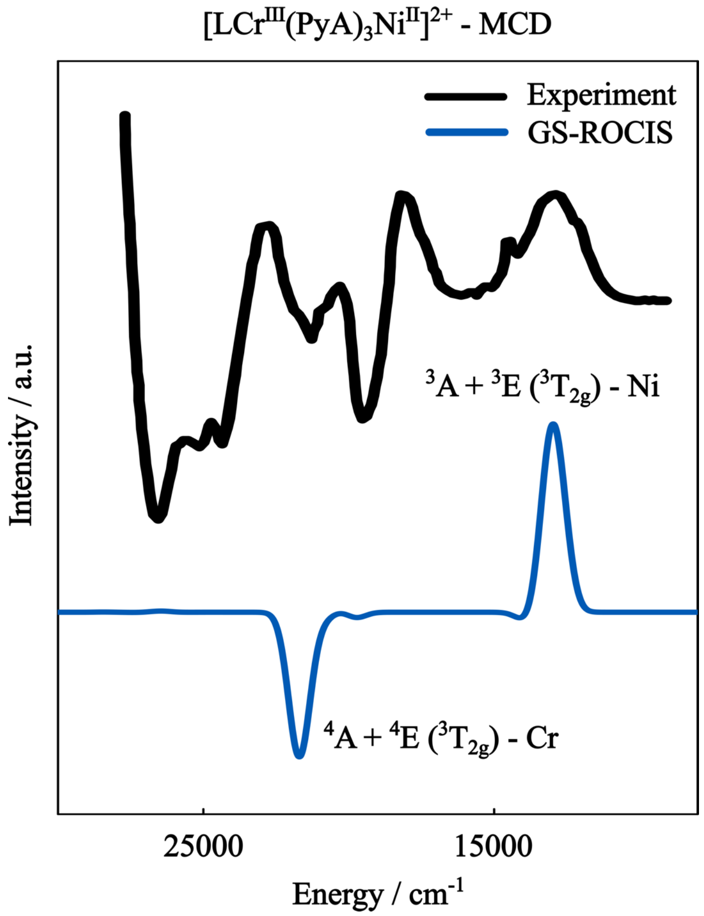


Figure S3: Experimental and GS-ROCIS calculated MCD spectrum of $[LCr^{III}(PyA)_3Ni^{II}]^{2+}$. Experimental data was digitized from reference 1. Calculated spectrum was shifted by −2700 $cm^{-1}$ and a Gaussian broadening of 800 $cm^{-1}$ was used.

# 5 $[Cu(H_2O)_6]^{2+}$

## 5.1 Molecular Geometry – XYZ (Å)

| | | | |
|---|---|---|---|
| Cu | 0.02449676034639 | 0.12459107191454 | -0.01922244949071 |
| O | -0.04945173837047 | 2.14812596448547 | -0.12945331858099 |
| H | 0.08620101197690 | 2.50523496532074 | -1.03044839798651 |
| H | 0.43641349954691 | 2.73024824495693 | 0.48686018762607 |
| O | 2.31498096381570 | -0.01389532719727 | -0.31835154676475 |
| H | 2.77840626509731 | -0.86816185777144 | -0.23704932032351 |
| H | 3.01744220311579 | 0.65001187043413 | -0.44697797882359 |
| O | 0.09847404126793 | -1.89834871726948 | 0.08925931726089 |
| H | -0.03268003602699 | -2.25765039238232 | 0.99008455320037 |
| H | -0.38958759307217 | -2.48022691395547 | -0.52559922539850 |
| O | -2.26619676346298 | 0.26089472991461 | 0.28095233821548 |
| H | -2.73297918710302 | 1.11301386584402 | 0.19623182059058 |
| H | -2.96624390265149 | -0.40514742836864 | 0.41168921103297 |
| O | 0.16200417511779 | 0.08179771156726 | 2.00517729978404 |
| H | 0.98492295640519 | 0.32244507618395 | 2.47469211516477 |
| H | -0.59533580203708 | 0.43645965691218 | 2.51236425688883 |
| O | -0.11067063104250 | 0.16892957594133 | -2.04388363355876 |
| H | 0.64645552140310 | -0.18624540265186 | -2.55098570629814 |
| H | -0.93307174432632 | -0.06686669387868 | -2.51674952253856 |

## 5.2 Excited states obtained with GS-ROCIS

Table S1: Energies and CSF composition of the non-relativistic ground and excited states resulting from excitations of the Cu $2p$ core orbitals of $[Cu(H_2O)_6]^{2+}$ obtained with GS-ROCIS. The single excited CSFs are represented as $|\Phi_p^q\rangle$, corresponding to the orbital excitation $p \rightarrow q$, while the reference CSF is represented as $|\Phi_0\rangle$.

| State | Composition | Energy / eV |
|---|---|---|
| $\|\boldsymbol{\Psi_0}\rangle(\,^2\boldsymbol{B_{1g}})$ | 100% $\|\Phi_0\rangle$ | 0 |
| $\|\boldsymbol{\Psi_1}\rangle(\,^2\boldsymbol{E_u})$ | 93%$\left\|\Phi_{2p_x}^{3d_{x^2-y^2}}\right\rangle$, 4%$\left\|\Phi_{2p_y}^{3d_{x^2-y^2}}\right\rangle$ | 937.06 |
| $\|\boldsymbol{\Psi_2}\rangle(\,^2\boldsymbol{E_u})$ | 4%$\left\|\Phi_{2p_x}^{3d_{x^2-y^2}}\right\rangle$, 93%$\left\|\Phi_{2p_y}^{3d_{x^2-y^2}}\right\rangle$ | 937.08 |
| $\|\boldsymbol{\Psi_3}\rangle(\,^2\boldsymbol{A_{2u}})$ | 98%$\left\|\Phi_{2p_z}^{3d_{x^2-y^2}}\right\rangle$ | 937.53 |

Table S2: Energies of the calculated SOC states and composition in relation to the non-relativistic states shown in Table S1.

| State | Composition | Energy/ eV |
|---|---|---|
| $\boldsymbol{E'_3(\Gamma_7)}$ | $100\%\lvert\Psi_0\rangle\left(M_s=-\frac{1}{2}\right)$ | 0 |
| $\boldsymbol{E'_3(\Gamma_7)}$ | $100\%\lvert\Psi_0\rangle\left(M_s=\frac{1}{2}\right)$ | 0 |
| $\boldsymbol{E'_3(\Gamma_7)}$ | $10\%\lvert\Psi_1\rangle\left(M_s=\frac{1}{2}\right)$, $41\%\lvert\Psi_1\rangle\left(M_s=-\frac{1}{2}\right)$,<br>$41\%\lvert\Psi_2\rangle\left(M_s=\frac{1}{2}\right)$, $8\%\lvert\Psi_2\rangle\left(M_s=-\frac{1}{2}\right)$ | 930.67 |
| $\boldsymbol{E'_3(\Gamma_7)}$ | $41\%\lvert\Psi_1\rangle\left(M_s=\frac{1}{2}\right)$, $10\%\lvert\Psi_1\rangle\left(M_s=-\frac{1}{2}\right)$,<br>$8\%\lvert\Psi_2\rangle\left(M_s=\frac{1}{2}\right)$, $41\%\lvert\Psi_2\rangle\left(M_s=-\frac{1}{2}\right)$ | 930.67 |
| $\boldsymbol{E'_2(\Gamma_6)}$ | $16\%\lvert\Psi_1\rangle\left(M_s=-\frac{1}{2}\right)$, $18\%\lvert\Psi_2\rangle\left(M_s=\frac{1}{2}\right)$,<br>$8\%\lvert\Psi_3\rangle\left(M_s=\frac{1}{2}\right)$, $58\%\lvert\Psi_3\rangle\left(M_s=-\frac{1}{2}\right)$ | 930.97 |
| $\boldsymbol{E'_2(\Gamma_6)}$ | $16\%\lvert\Psi_1\rangle\left(M_s=\frac{1}{2}\right)$, $18\%\lvert\Psi_2\rangle\left(M_s=-\frac{1}{2}\right)$,<br>$58\%\lvert\Psi_3\rangle\left(M_s=\frac{1}{2}\right)$, $8\%\lvert\Psi_3\rangle\left(M_s=-\frac{1}{2}\right)$ | 930.97 |
| $\boldsymbol{E'_2(\Gamma_6)}$ | $6\%\lvert\Psi_1\rangle\left(M_s=\frac{1}{2}\right)$, $27\%\lvert\Psi_1\rangle\left(M_s=-\frac{1}{2}\right)$,<br>$26\%\lvert\Psi_2\rangle\left(M_s=\frac{1}{2}\right)$, $7\%\lvert\Psi_2\rangle\left(M_s=-\frac{1}{2}\right)$,<br>$13\%\lvert\Psi_3\rangle\left(M_s=\frac{1}{2}\right)$, $21\%\lvert\Psi_3\rangle\left(M_s=-\frac{1}{2}\right)$ | 950.05 |
| $\boldsymbol{E'_2(\Gamma_6)}$ | $27\%\lvert\Psi_1\rangle\left(M_s=\frac{1}{2}\right)$, $6\%\lvert\Psi_1\rangle\left(M_s=-\frac{1}{2}\right)$,<br>$7\%\lvert\Psi_2\rangle\left(M_s=\frac{1}{2}\right)$, $26\%\lvert\Psi_2\rangle\left(M_s=-\frac{1}{2}\right)$,<br>$21\%\lvert\Psi_3\rangle\left(M_s=\frac{1}{2}\right)$, $13\%\lvert\Psi_3\rangle\left(M_s=-\frac{1}{2}\right)$ | 950.05 |

# 6 [$Cu_2(OAc)_4(H_2O)_2$]

## 6.1 Molecular Geometry – XYZ (Å)

| | | | |
|---|---|---|---|
| Cu | -0.89889381198418 | -0.15679012661551 | 0.23025366913522 |
| Cu | 1.57930276890720 | -0.13262969818937 | -0.10982374575458 |
| O | 3.84561703399200 | 0.38275720440372 | 0.01987077822517 |
| H | 3.67722005650988 | 1.32433244520985 | -0.17483047876096 |
| H | 3.82657981481919 | 0.33679487097671 | 0.99492407348044 |
| O | -3.18683058965429 | 0.19606622897230 | -0.00210154974900 |
| H | -3.08713714270172 | 1.16618990044432 | 0.03295410310509 |
| H | -3.13705502612929 | -0.01180008359700 | -0.95502057956310 |
| O | -0.83138502523070 | 1.85155767295318 | 0.02427162116704 |
| O | 1.41248261476777 | 1.87498973439611 | -0.27611648103131 |
| O | -0.74115807920295 | -2.12367125542438 | 0.38200905241165 |
| O | 1.51340440876036 | -2.09851440723437 | 0.10455900350995 |
| C | 0.39815722657869 | -2.68363665890191 | 0.28917667930136 |
| C | 0.27833381540688 | 2.44217941912013 | -0.17108700364935 |
| C | 0.25358107332338 | 3.95336742395291 | -0.25887393948161 |
| H | -0.73461826406373 | 4.30833115830455 | -0.57130168926868 |
| H | 1.02864952265482 | 4.31025045887837 | -0.94693417183087 |
| H | 0.46897042674042 | 4.36266661153088 | 0.73939999360633 |
| C | 0.42501665061255 | -4.19402683925202 | 0.38148922998109 |
| H | 1.40502060939189 | -4.54063461674788 | 0.72738996406797 |
| H | 0.24594156939902 | -4.60586552583248 | -0.62273434764423 |
| H | -0.37276456180673 | -4.55008242093537 | 1.04322681358892 |
| O | 1.23421468520749 | -0.31234202951020 | -2.05513601458490 |
| O | -1.01616572898695 | -0.32690500827016 | -1.77695272424697 |
| C | 0.04157321180558 | -0.37495339442282 | -2.48742568019539 |
| C | -0.14705580401310 | -0.53453313486794 | -3.98084947507759 |
| H | -0.54664703896151 | -1.53869257798006 | -4.18247880774910 |
| H | 0.80364787869295 | -0.40956661637282 | -4.50888947168234 |
| H | -0.88448610821772 | 0.19339346677208 | -4.34311790830258 |
| O | -0.54963715302712 | 0.04053263023768 | 2.17314879513377 |
| O | 1.70035927900166 | 0.07799567758897 | 1.89154625789970 |
| C | 0.64341610265112 | 0.10747442310978 | 2.60408600698794 |
| C | 0.82997613075602 | 0.20627149679869 | 4.10287266333394 |
| H | 1.67112279709038 | 0.86880024726326 | 4.33984296730455 |
| H | 1.07027886802407 | -0.79503727015650 | 4.48966744859608 |
| H | -0.08836321111333 | 0.55750159339729 | 4.58514394773633 |

## 6.2 Molecular orbitals obtained with CSF-ROHF

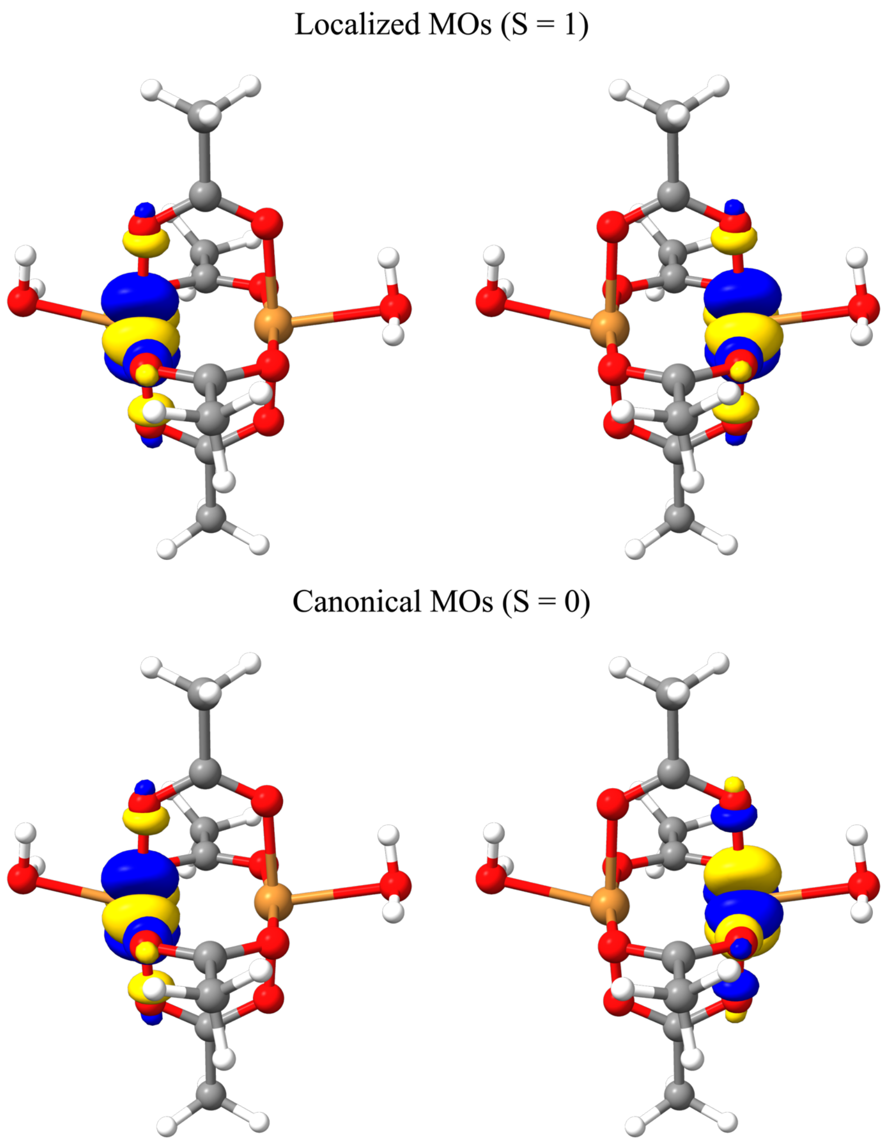


Figure S4: Localized molecular orbitals obtained from the high-spin ROHF calculation of $[Cu_2(OAc)_4(H_2O)_6]$ in the $S = 1$ spin state (top) and canonical molecular orbitals obtained from the CSF-ROHF calculation of the $S = 0$ state (bottom).

### 6.3 Excited state obtained with GS-ROCIS

Table S3: Energies (in eV) and CSF composition of the non-relativistic states of $[Cu_2(OAc)_4(H_2O)_6]$ calculated with GS-ROCIS for both magnetic coupling situations. The single excited CSFs are represented as $|\Phi_p^q\rangle$, corresponding to the orbital excitation $p \rightarrow q$, while the reference CSF is represented as $|\Phi_0\rangle$.

| **Ferromagnetic ($S = 1$)** | | | **Antiferromagnetic ($S = 0$)** | | |
|---|---|---|---|---|---|
| **State** | Composition | Energy | **State** | Composition | Energy |
| $|\Psi_0^3\rangle$ | 100% $|\Phi_0\rangle$ | 0 | $|\Psi_0^1\rangle$ | 100% $|\Phi_0\rangle$ | 0 |

| | | | | | |
|---|---|---|---|---|---|
| $\left\vert\boldsymbol{\Psi}_1^3\right\rangle$ | $93\%\left\vert\Phi_{2p_x}^{3d_{x^2-y^2}}\right\rangle$, $4\%\left\vert\Phi_{2p_y}^{3d_{x^2-y^2}}\right\rangle$ | 937.57 | $\left\vert\boldsymbol{\Psi}_1^3\right\rangle$ | $93\%\left\vert\Phi_{2p_x}^{3d_{x^2-y^2},+}\right\rangle$, $4\%\left\vert\Phi_{2p_y}^{3d_{x^2-y^2},+}\right\rangle$ | 937.57 |
| $\left\vert\boldsymbol{\Psi}_1^1\right\rangle$ | $93\%\left\vert\Phi_{2p_x}^{3d_{x^2-y^2},-}\right\rangle$, $4\%\left\vert\Phi_{2p_y}^{3d_{x^2-y^2},-}\right\rangle$ | 937.57 | $\left\vert\boldsymbol{\Psi}_1^1\right\rangle$ | $93\%\left\vert\Phi_{2p_x}^{3d_{x^2-y^2}}\right\rangle$, $4\%\left\vert\Phi_{2p_y}^{3d_{x^2-y^2}}\right\rangle$ | 937.57 |
| $\left\vert\boldsymbol{\Psi}_2^3\right\rangle$ | $4\%\left\vert\Phi_{2p_x}^{3d_{x^2-y^2}}\right\rangle$, $93\%\left\vert\Phi_{2p_y}^{3d_{x^2-y^2}}\right\rangle$ | 937.58 | $\left\vert\boldsymbol{\Psi}_2^3\right\rangle$ | $4\%\left\vert\Phi_{2p_x}^{3d_{x^2-y^2},+}\right\rangle$, $93\%\left\vert\Phi_{2p_y}^{3d_{x^2-y^2},+}\right\rangle$ | 937.58 |
| $\left\vert\boldsymbol{\Psi}_2^1\right\rangle$ | $4\%\left\vert\Phi_{2p_x}^{3d_{x^2-y^2},-}\right\rangle$, $93\%\left\vert\Phi_{2p_y}^{3d_{x^2-y^2},-}\right\rangle$ | 937.58 | $\left\vert\boldsymbol{\Psi}_2^1\right\rangle$ | $4\%\left\vert\Phi_{2p_x}^{3d_{x^2-y^2}}\right\rangle$, $93\%\left\vert\Phi_{2p_y}^{3d_{x^2-y^2}}\right\rangle$ | 937.58 |
| $\left\vert\boldsymbol{\Psi}_3^3\right\rangle$ | $97\%\left\vert\Phi_{2p_z}^{3d_{x^2-y^2}}\right\rangle$ | 938.13 | $\left\vert\boldsymbol{\Psi}_3^3\right\rangle$ | $97\%\left\vert\Phi_{2p_z}^{3d_{x^2-y^2},+}\right\rangle$ | 938.13 |
| $\left\vert\boldsymbol{\Psi}_3^1\right\rangle$ | $97\%\left\vert\Phi_{2p_z}^{3d_{x^2-y^2},-}\right\rangle$ | 938.14 | $\left\vert\boldsymbol{\Psi}_3^1\right\rangle$ | $97\%\left\vert\Phi_{2p_z}^{3d_{x^2-y^2}}\right\rangle$ | 938.13 |

### 6.4 GS-ROCIS Calculated $L_{2,3}$-edge XMCD

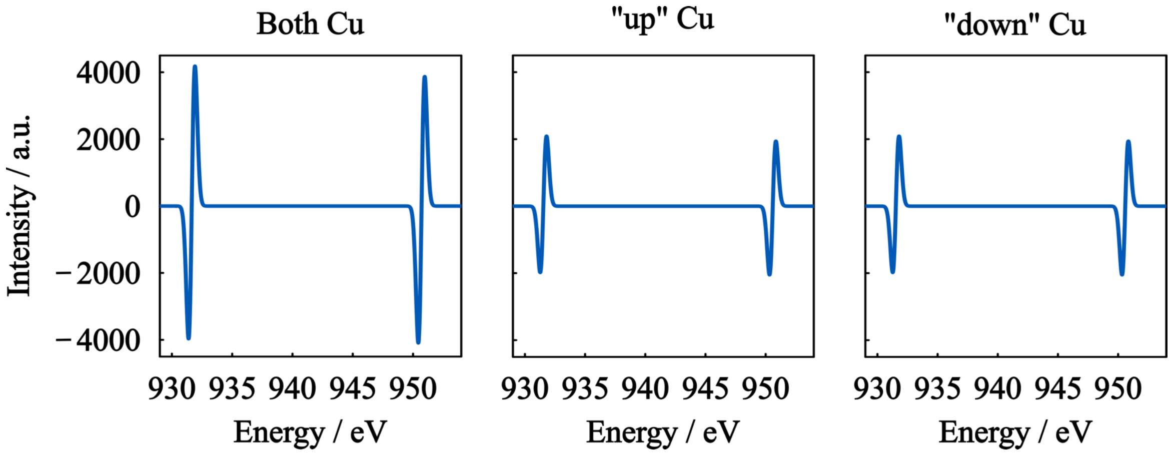


Figure S5: GS-ROCIS calculated $L_{2,3}$-edge XMCD spectra of $[Cu_2(OAc)_4(H_2O)_2]$ in the $S = 0$ state, for orbital spaces containing only one of the Cu centers, and both Cu center $2p$ orbitals as donor orbitals.

# 7 [$(F_8TPP)Fe(\mu\text{-}O)Cu(TMPA)]^+$

## 7.1 Molecular Geometry – XYZ (Å)

| | | | |
|---|---|---|---|
| Cu | 12.73582000000000 | 17.14475000000000 | -0.18495000000000 |
| Fe | 9.14597000000000 | 17.33560000000000 | -0.10924000000000 |
| O | 10.88324000000000 | 17.25686000000000 | -0.17151000000000 |
| N | 14.83660000000000 | 17.13910999999999 | -0.18387000000000 |
| N | 13.03307000000000 | 17.28629000000000 | 1.79695000000000 |
| N | 13.05909000000000 | 14.99764000000000 | -0.09116000000000 |
| N | 13.01505000000000 | 17.48008000000000 | -2.11988000000000 |
| N | 8.75343000000000 | 19.41303999999999 | -0.03399000000000 |
| N | 8.59730000000000 | 17.28384000000000 | 1.92056000000000 |
| N | 8.47720000000000 | 15.34352000000000 | -0.14678000000000 |
| N | 8.54726000000000 | 17.47517000000000 | -2.11679000000000 |
| F | 6.94790000000000 | 21.49319000000000 | 3.33278000000000 |
| F | 11.30360000000000 | 19.94780000000000 | 3.78704000000000 |
| F | 6.11519000000000 | 13.25356000000000 | 2.80745000000000 |
| F | 10.57498000000000 | 14.13173000000000 | 3.86120000000000 |
| F | 6.14322000000000 | 13.63377000000000 | -3.52592000000000 |
| F | 10.73311000000000 | 14.25929000000000 | -3.97400000000000 |
| F | 6.72771000000000 | 21.87831000000000 | -2.88470000000000 |
| F | 10.89725000000000 | 20.17591999999999 | -4.17640000000000 |
| C | 12.06024000000000 | 17.04835000000000 | 2.70547000000000 |
| C | 12.36650000000000 | 16.97967000000000 | 4.06825000000000 |
| C | 13.68562000000000 | 17.08759999999999 | 4.46997000000000 |
| C | 14.60641000000000 | 17.34515999999999 | 3.53055000000000 |
| C | 14.26411000000000 | 17.41875000000000 | 2.19250000000000 |
| C | 15.24094000000000 | 17.74255000000000 | 1.10784000000000 |
| C | 12.18235000000000 | 14.06060000000000 | 0.12361000000000 |
| C | 12.56267000000000 | 12.76787000000000 | 0.38627000000000 |
| C | 13.93584000000000 | 12.52747000000000 | 0.61804000000000 |
| C | 14.78856000000000 | 13.46452000000000 | 0.44808000000000 |
| C | 14.37221000000000 | 14.70819000000000 | 0.07107000000000 |
| C | 15.30700000000000 | 15.72864000000000 | -0.33220000000000 |
| C | 12.07626000000000 | 17.35252000000000 | -3.04539000000000 |
| C | 12.37451000000000 | 17.44819000000000 | -4.41744000000000 |
| C | 13.66961000000000 | 17.62725999999999 | -4.79599000000000 |
| C | 14.68047000000000 | 17.76463000000000 | -3.78704000000000 |
| C | 14.30815000000000 | 17.71066000000000 | -2.48143000000000 |
| C | 15.25095000000000 | 17.92161999999999 | -1.37359000000000 |
| C | 8.79347000000000 | 20.25933000000000 | -1.10784000000000 |
| C | 8.95761000000000 | 21.61338000000000 | -0.61340000000000 |
| C | 9.05369000000000 | 21.55696000000000 | 0.71538000000000 |
| C | 8.91157000000000 | 20.17591999999999 | 1.08621000000000 |
| C | 8.88154000000000 | 19.70250000000000 | 2.40572000000000 |
| C | 8.67737000000000 | 18.37542000000000 | 2.77036000000000 |
| C | 8.51723000000000 | 17.91915999999999 | 4.14396000000000 |

| | | | |
|---|---|---|---|
| C | 8.38912000000000 | 16.57002000000000 | 4.09606000000000 |
| C | 8.41515000000000 | 16.17508000000000 | 2.70702000000000 |
| C | 8.37711000000000 | 14.86763000000000 | 2.25430000000000 |
| C | 8.43116000000000 | 14.47515000000000 | 0.91161000000000 |
| C | 8.33308000000000 | 13.11864000000000 | 0.41100000000000 |
| C | 8.32907000000000 | 13.17506000000000 | -0.91470000000000 |
| C | 8.41315000000000 | 14.55120000000000 | -1.29170000000000 |
| C | 8.45518000000000 | 15.03444000000000 | -2.61431000000000 |
| C | 8.47320000000000 | 16.37132000000000 | -2.97277000000000 |
| C | 8.32307000000000 | 16.86928000000000 | -4.33092000000000 |
| C | 8.41114000000000 | 18.21353000000000 | -4.28302000000000 |
| C | 8.55527000000000 | 18.60355000000000 | -2.89706000000000 |
| C | 8.72541000000000 | 19.89138000000000 | -2.42426000000000 |
| C | 9.11774000000000 | 20.67634000000000 | 3.51201000000000 |
| C | 8.16694000000000 | 21.53242999999999 | 3.93073000000000 |
| C | 8.33908000000000 | 22.47929000000000 | 4.94432000000000 |
| C | 9.58214000000000 | 22.45721999999999 | 5.56236000000000 |
| C | 10.59900000000000 | 21.65507999999999 | 5.18072000000000 |
| C | 10.32677000000000 | 20.77691000000000 | 4.14396000000000 |
| C | 8.33908000000000 | 13.77605000000000 | 3.29261000000000 |
| C | 9.42600000000000 | 13.44489000000000 | 4.11306000000000 |
| C | 9.43601000000000 | 12.50049000000000 | 5.05402000000000 |
| C | 8.32307000000000 | 11.76949000000000 | 5.26261000000000 |
| C | 7.13406000000000 | 12.02215000000000 | 4.51324000000000 |
| C | 7.20812000000000 | 13.01562000000000 | 3.55682000000000 |
| C | 8.43116000000000 | 14.00908000000000 | -3.71906000000000 |
| C | 7.32222000000000 | 13.35904000000000 | -4.15941000000000 |
| C | 7.33423000000000 | 12.41463000000000 | -5.16527000000000 |
| C | 8.48120000000000 | 12.13990000000000 | -5.77404000000000 |
| C | 9.66421000000000 | 12.69918000000000 | -5.38622000000000 |
| C | 9.59215000000000 | 13.65830000000000 | -4.34637000000000 |
| C | 8.82149000000000 | 20.96579000000000 | -3.45948000000000 |
| C | 7.86068000000000 | 21.92491000000000 | -3.67425000000000 |
| C | 7.96276000000000 | 22.96744000000000 | -4.57504000000000 |
| C | 9.13376000000000 | 23.00178000000000 | -5.33523000000000 |
| C | 10.12460000000000 | 22.09171999999999 | -5.20390000000000 |
| C | 9.94244000000000 | 21.08107999999999 | -4.28611000000000 |
| H | 8.97562000000000 | 22.41306000000000 | -1.17737000000000 |
| H | 9.21783000000000 | 22.33457000000000 | 1.29479000000000 |
| H | 8.52924000000000 | 18.49317000000000 | 4.93814000000000 |
| H | 8.27903000000000 | 15.97148000000000 | 4.85625000000000 |
| H | 8.33908000000000 | 12.27726000000000 | 0.95796000000000 |
| H | 8.21498000000000 | 12.41954000000000 | -1.52347000000000 |
| H | 8.17494000000000 | 16.31736000000000 | -5.14364000000000 |
| H | 8.34108000000000 | 18.82677999999999 | -5.05866000000000 |
| H | 7.64649000000000 | 23.12198000000000 | 5.23171000000000 |
| H | 9.71425000000000 | 23.09500000000000 | 6.32255000000000 |
| H | 11.44372000000000 | 21.75075000000000 | 5.65661000000000 |
| H | 10.24270000000000 | 12.30670000000000 | 5.57318000000000 |
| H | 8.31907000000000 | 11.05077000000000 | 5.92391000000000 |

| | | | |
|---|---|---|---|
| H | 6.31536000000000 | 11.49230000000000 | 4.65539000000000 |
| H | 6.52354000000000 | 11.89950000000000 | -5.41403000000000 |
| H | 8.47720000000000 | 11.53155000000000 | -6.54350000000000 |
| H | 10.54696000000000 | 12.43426000000000 | -5.81576000000000 |
| H | 7.23414000000000 | 23.63956000000000 | -4.70483000000000 |
| H | 9.24986000000000 | 23.73523000000000 | -5.97181000000000 |
| H | 10.91127000000000 | 22.21682000000000 | -5.71069000000000 |
| H | 11.10743000000000 | 16.98457000000000 | 2.41190000000000 |
| H | 11.66591000000000 | 16.84966000000000 | 4.74191000000000 |
| H | 13.93784000000000 | 16.97967000000000 | 5.40012000000000 |
| H | 15.54120000000000 | 17.50460999999999 | 3.79940000000000 |
| H | 16.12770000000000 | 17.46780999999999 | 1.36278000000000 |
| H | 15.28899000000000 | 18.72865000000000 | 1.02131000000000 |
| H | 11.23354000000000 | 14.28382000000000 | 0.11125000000000 |
| H | 11.89210000000000 | 12.05404000000000 | 0.41409000000000 |
| H | 14.23209000000000 | 11.59533000000000 | 0.85753000000000 |
| H | 15.74938000000000 | 13.29281000000000 | 0.59950000000000 |
| H | 15.53319000000000 | 15.58882000000000 | -1.26235000000000 |
| H | 16.13370000000000 | 15.63787000000000 | 0.16687000000000 |
| H | 11.17949000000000 | 17.14156000000000 | -2.74873000000000 |
| H | 11.62587000000000 | 17.37705000000000 | -5.08492000000000 |
| H | 13.92783000000000 | 17.64688000000000 | -5.74777000000000 |
| H | 15.29099000000000 | 18.86357000000000 | -1.13874000000000 |
| H | 16.14571000000000 | 17.66405000000000 | -1.62390000000000 |
| H | 15.60325000000000 | 17.93143000000000 | -4.02035000000000 |

## 7.2 Molecular orbitals obtained from CSF-ROHF

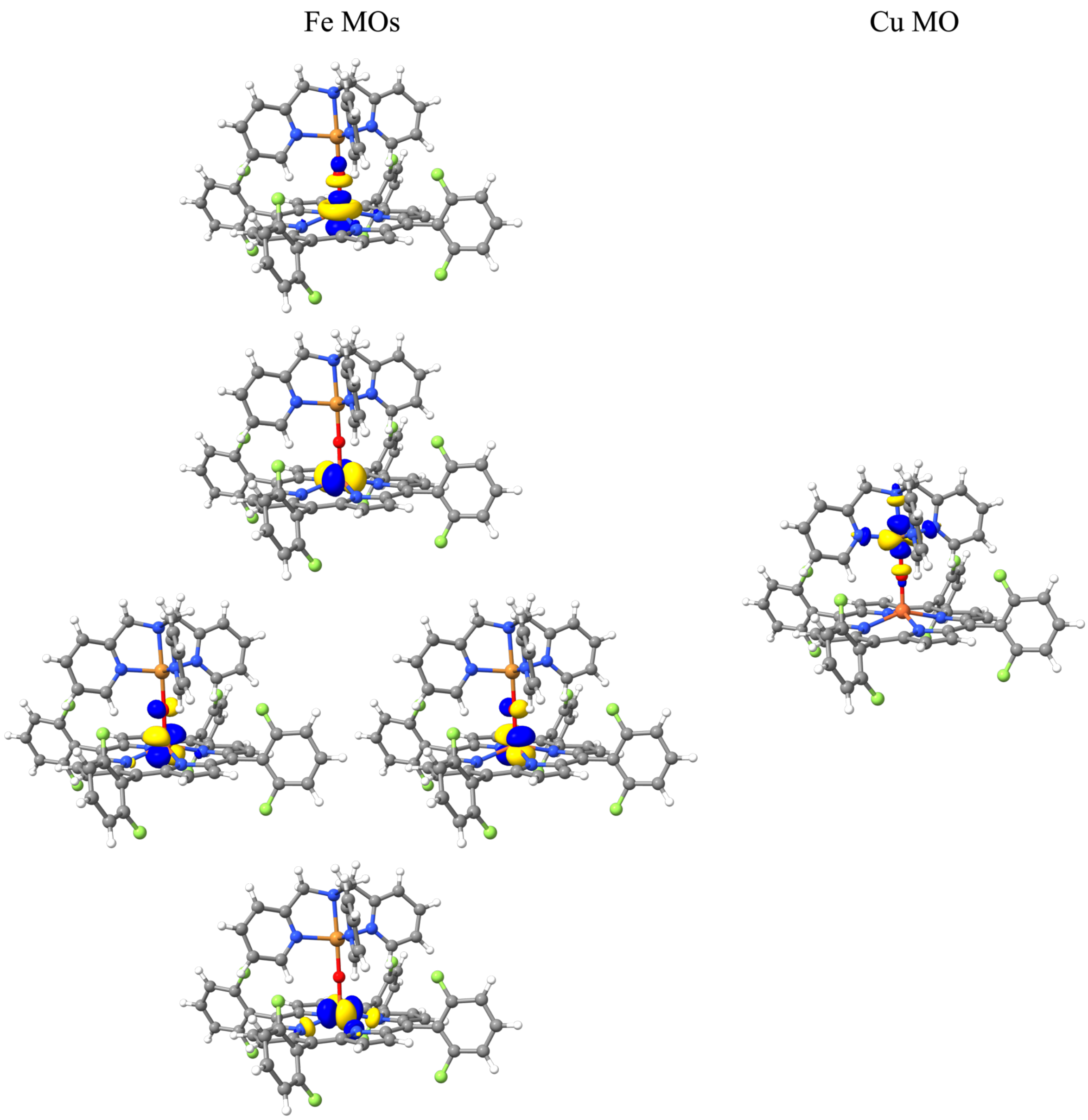


Figure S6: Canonical molecular orbitals obtained from the CSF-ROHF calculation of $[(F_8TPP)Fe(\mu\text{-}O)Cu(TMPA)]^+$ in the $S = 2$ spin state.

# 8 $[Fe^{II}(SPh)_4]^{2-}$

## 8.1 Molecular Geometry – XYZ (Å)

| | | | |
|---|---|---|---|
| Fe | -1.12871468040694 | -0.66104549069796 | 0.25244925035827 |
| S | 0.86938766755235 | -1.54320375325019 | -0.55920053734843 |
| S | -0.87782536765301 | 0.67918235430013 | 2.13563006865059 |
| S | -2.25511600584005 | -2.42224142783124 | 1.20672614628107 |
| S | -2.57594969234484 | 0.18743995242487 | -1.39311429246093 |
| C | -0.57322747245635 | 2.35593213641430 | 1.71041500293940 |
| C | -1.03035439786330 | 2.93824490794105 | 0.50384345191445 |
| C | -0.74069103124675 | 4.26367014444229 | 0.18747484485218 |
| C | -0.00252184189041 | 5.07157611661770 | 1.06312702241580 |
| C | 0.43854079117119 | 4.51730304699649 | 2.27235641216368 |
| C | 0.16064100839996 | 3.18840962277909 | 2.58961618679779 |
| H | 0.52652349254389 | 2.75605944447832 | 3.52411890055383 |
| H | -1.61194747513918 | 2.31558968475446 | -0.18036959624927 |
| H | -1.08843967978946 | 4.66653348798451 | -0.76773885798764 |
| H | 0.23040608230153 | 6.10786535172035 | 0.80614741206355 |
| H | 1.02197176336273 | 5.12546641592645 | 2.97121359987936 |
| C | 1.80196022788401 | -0.24969905101571 | -1.29224895364269 |
| C | 1.51976779388315 | 1.11402474913987 | -1.05735739369494 |
| C | 2.23628075171094 | 2.12220042159853 | -1.69322445988973 |
| C | 3.27583607876912 | 1.81246588001509 | -2.57997164057503 |
| C | 3.58427042909728 | 0.46609250670360 | -2.80913582033137 |
| C | 2.86527461022827 | -0.54738749334815 | -2.17459163353763 |
| H | 0.71169831072143 | 1.37436055587166 | -0.37510653669381 |
| H | 1.96406891908813 | 3.16203074440458 | -1.49809757192950 |
| H | 3.82588833029576 | 2.60480709041810 | -3.09326220231481 |
| H | 4.38946039696013 | 0.20004963171535 | -3.50179454857285 |
| H | 3.09907804584038 | -1.59635994140595 | -2.37283602143253 |
| C | -2.55088022263834 | -3.73376368640349 | 0.07677877463574 |
| C | -3.22794733251028 | -4.89944998886105 | 0.50989282432653 |
| C | -3.48371359686585 | -5.96046204750168 | -0.35816900385742 |
| C | -3.07567089959720 | -5.89995121128803 | -1.69726885480571 |
| C | -2.40650579222550 | -4.75024835382251 | -2.14128600504432 |
| C | -2.14733137044130 | -3.68822908142080 | -1.27828997342001 |
| H | -3.54998916720559 | -4.95092084766070 | 1.55332283485370 |
| H | -4.00872327835913 | -6.84635848858120 | 0.01421370912330 |
| H | -3.27475679283173 | -6.73006692212358 | -2.37984150469554 |
| H | -2.07865840310760 | -4.67863319567591 | -3.18246972024358 |
| H | -1.62631247134060 | -2.79788266086611 | -1.63306380648820 |
| C | -1.56201640716485 | 0.87291851934670 | -2.66132693309893 |
| C | -1.68224971083406 | 2.23564142149487 | -3.01185063724479 |
| C | -0.87724598955876 | 2.80446309287443 | -3.99950511937769 |
| C | 0.07103715908284 | 2.03044639631223 | -4.67584395172593 |
| C | 0.19092529861986 | 0.67439134609019 | -4.35072386277842 |
| C | -0.61155375478289 | 0.10324775425423 | -3.36650610660027 |

| | | | |
|---|---|---|---|
| H | -0.47709604578828 | -0.94109212843214 | -3.08426865745868 |
| H | 0.72709714023848 | 2.48132229901622 | -5.42379430898250 |
| H | 0.94868178672772 | 0.06009491982099 | -4.84204625208451 |
| H | -2.41253219882550 | 2.84630720429276 | -2.47658908501450 |
| H | -0.97810500577137 | 3.86977857003695 | -4.22817259222693 |

# 9 $[Fe^{III}(SDur)_4]^-$

## 9.1 Molecular Geometry – XYZ (Å)

| | | | |
|---|---|---|---|
| Fe | 0.00457205836064 | 0.00253600836811 | 0.00153388597817 |
| S | 1.79525022270204 | -0.63333479337526 | 1.28317129752483 |
| C | 2.59599564121911 | 0.95143225672002 | 1.39722954696385 |
| C | 3.54755187710035 | 1.31131499328572 | 0.41375699279011 |
| C | 4.09099179989897 | 2.60729839079875 | 0.44170119621122 |
| C | 3.70035400273961 | 3.49099326855724 | 1.45061103714818 |
| C | 2.79061452433807 | 3.13267696121266 | 2.44938475852919 |
| C | 2.23158665068906 | 1.84218045114171 | 2.43519760348417 |
| C | 3.92244234313410 | 0.36016675046649 | -0.68950885264825 |
| C | 5.04682128300896 | 3.07227150850007 | -0.62775008495218 |
| C | 2.40618615560086 | 4.15404087793711 | 3.49272640551685 |
| C | 1.23264741452181 | 1.41371721592830 | 3.47314642665130 |
| S | 0.63318347180265 | 1.79931997082073 | -1.27528195608074 |
| S | -1.78781898344999 | 0.63054246804367 | 1.28517955341799 |
| S | -0.62965293408533 | -1.78431119119645 | -1.28619234300654 |
| C | -0.95303745616354 | 2.59611422401971 | -1.39485288504200 |
| C | -2.58998383178813 | -0.95309866462092 | 1.40122956920730 |
| C | 0.95266631397784 | -2.58966542290409 | -1.40022756459874 |
| C | -1.31892527447995 | 3.54543932202582 | -0.41134628376095 |
| C | -1.83846523236548 | 2.23214521128848 | -2.43743563025302 |
| C | -3.54253423618534 | -1.31262375831782 | 0.41860264275805 |
| C | -2.22678714892422 | -1.84286456108177 | 2.44033211621522 |
| C | 1.30833842502636 | -3.54487775021701 | -0.41877414202386 |
| C | 1.84549566077401 | -2.22586554220322 | -2.43651981866248 |
| C | -2.61552057467013 | 4.08709432467897 | -0.44442610202336 |
| C | -0.37314166856647 | 3.91997692079298 | 0.69666477679579 |
| C | -3.12962683271035 | 2.78965377021844 | -2.45689865449632 |
| C | -1.40391571910505 | 1.23560285960357 | -3.47515423713933 |
| C | -4.08882658818255 | -2.60733891332614 | 0.44905990166673 |
| C | -3.91597716957026 | -0.36202568170227 | -0.68562959907269 |
| C | -2.78922415023353 | -3.13188846945426 | 2.45753660303546 |
| C | -1.22575864792295 | -1.41478511017936 | 3.47647275185216 |
| C | 2.60212116403479 | -4.09345437245903 | -0.44752490304372 |
| C | 0.35484718344745 | -3.91837855350874 | 0.68290546120923 |
| C | 3.13361848724488 | -2.79037024422480 | -2.45186298084047 |
| C | 1.42163092456555 | -1.22203750682852 | -3.47168937362388 |
| C | -3.49388233054275 | 3.69716793467207 | -1.45833389022350 |
| C | -3.08706576857796 | 5.04054950945198 | 0.62432056287560 |
| C | -4.14504434750829 | 2.40642640466182 | -3.50648436593498 |
| C | -3.70021366905673 | -3.48992287962100 | 1.45981346212414 |
| C | -5.04618108340773 | -3.07221539539934 | -0.61911804897918 |
| C | -2.40777444451311 | -4.15182949768213 | 3.50341698341082 |
| C | 3.48774389949649 | -3.70414324600383 | -1.45531260332807 |
| C | 3.06291855100339 | -5.05398224946780 | 0.61955023804820 |

| | | | |
|---|---|---|---|
| C | 4.15661937983925 | -2.40805309607687 | -3.49437718531769 |
| H | 4.11379224960132 | 4.50425943937720 | 1.45298224657772 |
| H | 4.97279987338046 | 0.48449460109155 | -0.98968952827073 |
| H | 3.30169389574518 | 0.53556788378608 | -1.58511284199710 |
| H | 3.75130517672902 | -0.68128792494693 | -0.39315256397706 |
| H | 5.97004809547602 | 2.47033888120895 | -0.65047261427021 |
| H | 5.33259509857058 | 4.12072347591714 | -0.46286970032010 |
| H | 4.59462372112238 | 2.99722952698670 | -1.62954585115485 |
| H | 2.92070376283707 | 5.10650274663337 | 3.30255134648409 |
| H | 2.67024992533906 | 3.82856973091737 | 4.51202528206300 |
| H | 1.32289870660796 | 4.35213470852224 | 3.49048903813009 |
| H | 1.49184297280365 | 0.42154615506708 | 3.87090058857535 |
| H | 0.22535036401888 | 1.30517161272345 | 3.03554451353124 |
| H | 1.17292384453236 | 2.12331806110607 | 4.30773344943843 |
| H | 0.67007235676911 | 3.75406643227208 | 0.40338818720199 |
| H | -0.54912587901454 | 3.29488422325623 | 1.58914814475730 |
| H | -0.50294884740816 | 4.96856547948431 | 1.00051111592347 |
| H | -2.10942363083812 | 1.17667057501583 | -4.31324813276658 |
| H | -1.29657397208482 | 0.22767454100027 | -3.03866875670556 |
| H | -0.41007228009131 | 1.49643380315830 | -3.86763779599361 |
| H | -4.96555377858444 | -0.48716220428365 | -0.98803032529909 |
| H | -3.29307794205389 | -0.53684704843910 | -1.57986429449629 |
| H | -3.74614070685514 | 0.67958278875980 | -0.38899991714221 |
| H | -1.48385210773327 | -0.42252823483910 | 3.87479073683509 |
| H | -0.21926156953874 | -1.30649022990775 | 3.03697365958016 |
| H | -1.16467149859950 | -2.12440806427427 | 4.31093360670437 |
| H | -0.68580509479048 | -3.74485467144066 | 0.38507343810302 |
| H | 0.53014814524921 | -3.29832846168277 | 1.57898223400725 |
| H | 0.47643496626030 | -4.96907297909771 | 0.98289752811672 |
| H | 2.13320049271400 | -1.16056877110466 | -4.30444868680691 |
| H | 1.31408117054412 | -0.21627994885034 | -3.03034088734537 |
| H | 0.42982573715815 | -1.47754947390541 | -3.87276613616360 |
| H | -4.50766532376909 | 4.10931049786469 | -1.46488908491585 |
| H | -4.13524674499219 | 5.32465699462588 | 0.45484858839914 |
| H | -2.48699934523398 | 5.96487170618572 | 0.65114815138314 |
| H | -3.01604930947966 | 4.58702287800005 | 1.62580929324133 |
| H | -3.81527582095504 | 2.67547422617177 | -4.52313769408762 |
| H | -5.09979098482391 | 2.91766503733093 | -3.31894534945412 |
| H | -4.34028033292318 | 1.32264560810820 | -3.50994130567032 |
| H | -4.11629263943769 | -4.50210505249328 | 1.46447531589598 |
| H | -5.96879183482631 | -2.46932578864334 | -0.64154485579117 |
| H | -5.33300658483529 | -4.12018519488236 | -0.45291555020792 |
| H | -4.59480199500675 | -2.99873739797431 | -1.62137987174857 |
| H | -2.92437162390093 | -5.10351164385434 | 3.31496889950352 |
| H | -2.67200956985700 | -3.82341374467026 | 4.52173586156530 |
| H | -1.32496475011956 | -4.35254534870350 | 3.50284813888587 |
| H | 4.49927108257014 | -4.12182815043789 | -1.45849820414957 |
| H | 4.11038786874889 | -5.34314053928389 | 0.45428655103277 |
| H | 2.45760851183295 | -5.97506436487793 | 0.63927710597461 |
| H | 2.98915461256139 | -4.60443381505849 | 1.62262592117409 |

| | | | |
|---|---|---|---|
| H | 3.83002576147892 | -2.66886433481348 | -4.51416984356896 |
| H | 5.10696574415045 | -2.92684253423564 | -3.30522434338450 |
| H | 4.35921068843501 | -1.32560039521396 | -3.49031707575980 |

# 10 $[L_2Fe^{II,III}{}_2S_2]^{3-}$

## 10.1 Molecular Geometry – XYZ (Å)

| | | | |
|---|---|---|---|
| N | -1.35367004975977 | -0.21713526739233 | -0.18416557360437 |
| C | -2.68693021022245 | -0.04959048336618 | -0.43404360695482 |
| N | -3.05479191923139 | 1.17203320897656 | -0.85795505346520 |
| C | -1.86526402550478 | 1.87136871058780 | -0.89149110010750 |
| C | -0.79857815877244 | 1.00953095161860 | -0.48134178638423 |
| C | -1.59197214885606 | 3.19874228704757 | -1.25511893401636 |
| C | -0.26582258213206 | 3.63766940246957 | -1.20548553921521 |
| C | 0.77963024895079 | 2.77182518566436 | -0.81415743064189 |
| C | 0.53006509650799 | 1.44590609603780 | -0.45508054652225 |
| C | -3.72919926182510 | -1.11962627056220 | -0.21358978433750 |
| C | -3.31685614176199 | -2.54143556460493 | -0.51370572220093 |
| H | -4.54342258505796 | -0.89348783617475 | -0.91583350034206 |
| C | -4.32465331968491 | -1.02083529167355 | 1.19120822227875 |
| N | -2.07644597594172 | -3.05089868726137 | -0.24866411988931 |
| N | -4.20834472661202 | -3.40622067798051 | -1.02755544212642 |
| C | -3.49828793922511 | -4.58700103390001 | -1.11141060399366 |
| C | -2.16530648089955 | -4.37162114455281 | -0.63662589449009 |
| C | -3.88079049599802 | -5.85545343255975 | -1.57346641321156 |
| C | -2.92953657849967 | -6.87934816513311 | -1.55551767010281 |
| C | -1.61261721807697 | -6.64929890186498 | -1.09923976016733 |
| C | -1.20989207641608 | -5.39324327643333 | -0.64177469795910 |
| H | -2.40170258589478 | 3.86501662892395 | -1.56695043532116 |
| H | -0.02880279151169 | 4.67106532390442 | -1.47736099620640 |
| H | 1.33554930825352 | 0.76328078945896 | -0.18082546230488 |
| H | 1.80613882255907 | 3.14457706701479 | -0.78859117445133 |
| H | -4.89828915613387 | -6.02859003912516 | -1.93608757600530 |
| H | -3.20579853518452 | -7.87921921933506 | -1.90466659869529 |
| H | -0.89136274623191 | -7.46971884571924 | -1.10123851117838 |
| H | -0.18672084761904 | -5.20179755166637 | -0.31586465504570 |
| Fe | -0.46824948738798 | -1.96773930972724 | 0.39651409877453 |
| C | -3.53239623744737 | -1.20467739311685 | 2.33263878166119 |
| C | -4.09840745650734 | -1.11930812562688 | 3.60482429034037 |
| C | -5.46185872115610 | -0.84749957096367 | 3.76230861781010 |
| C | -6.25765919087729 | -0.66190012584382 | 2.62826402733015 |
| C | -5.68889804090754 | -0.74923858448983 | 1.35442416640376 |
| H | -6.30870514127772 | -0.60745364503782 | 0.46584986797723 |
| H | -5.90139974557947 | -0.78202925447507 | 4.76164693503060 |
| H | -7.32561532273092 | -0.44915061752083 | 2.73390226885113 |
| H | -2.46160838505306 | -1.42231704416313 | 2.24497098917815 |
| H | -3.45560092057231 | -1.27105867257470 | 4.47472811965608 |
| H | 1.23725411064905 | -9.18785209117863 | 3.48422268754815 |
| C | 1.49813379784891 | -8.16637778856654 | 3.19012450663924 |
| H | 3.62738567829335 | -8.43273055107661 | 3.56403676346089 |
| C | 2.83353726678330 | -7.75601339959133 | 3.23440438599944 |

| | | | |
|---|---|---|---|
| H | -0.56097938018089 | -7.63673020722245 | 2.75609540797741 |
| C | 0.47346496573808 | -7.28639854979941 | 2.77649337322038 |
| C | 3.13718260565442 | -6.44343437927912 | 2.84241068483488 |
| C | 0.75338438145964 | -5.97471012536110 | 2.38867813911056 |
| N | 4.34208394533012 | -5.77155836200667 | 2.79862738785889 |
| C | 2.09136586891414 | -5.56753103944378 | 2.40924763550882 |
| H | -0.03646018259278 | -5.28118266557417 | 2.09693753712341 |
| C | 4.00344760186989 | -4.55173632615980 | 2.34671298660512 |
| N | 2.67504479254973 | -4.36018486477613 | 2.08683980110820 |
| C | 6.77649002757249 | -3.88522894688600 | -1.87104249451302 |
| C | 7.56970521049980 | -4.08739763281487 | -0.73796928397098 |
| H | 7.20960092880432 | -3.97756962492411 | -2.87107357654763 |
| C | 5.42439381821862 | -3.56261083696095 | -1.71155152446606 |
| H | 8.62884611966684 | -4.33975956343542 | -0.84501871022127 |
| C | 7.00952083488295 | -3.96613023841570 | 0.53684082262560 |
| C | 4.86694196812848 | -3.44324896295499 | -0.43827557490555 |
| C | 5.65657008875215 | -3.64359181933490 | 0.70214539118468 |
| H | 4.78415251562569 | -3.39733637457737 | -2.58087266728357 |
| H | 7.62717000668003 | -4.12138787842562 | 1.42466611689131 |
| C | 5.07035115640008 | -3.51026976491338 | 2.10808040858586 |
| H | 3.80528638371036 | -3.18551738315554 | -0.34852480633364 |
| H | 5.88169763043212 | -3.74759977957495 | 2.80987854819702 |
| Fe | 1.82794957627186 | -2.59720323615579 | 1.49394844078807 |
| C | 4.69283895841461 | -2.07516019111114 | 2.39025192955958 |
| N | 3.46063143180119 | -1.54333285440329 | 2.13212487725427 |
| N | 5.60938369982564 | -1.22167208450953 | 2.87879639410366 |
| C | 3.58319927953905 | -0.21872304403610 | 2.49548543706603 |
| C | 4.92620226505757 | -0.02444096219060 | 2.95093310831103 |
| H | 1.61971336646442 | 0.64761134554267 | 2.18162388032348 |
| C | 2.65030982318193 | 0.82338427222008 | 2.49260630142507 |
| C | 5.34117698672859 | 1.24341895416273 | 3.38600327819188 |
| C | 3.08526823309534 | 2.07838371416767 | 2.92275675962323 |
| H | 6.36633784727231 | 1.40112174020610 | 3.73382852169105 |
| C | 4.41215333430630 | 2.28746435485163 | 3.36020437264875 |
| H | 2.38190659263218 | 2.91424531988899 | 2.91794493874003 |
| H | 4.71425260781756 | 3.28704732608299 | 3.68818847198905 |
| S | 1.43502865309967 | -2.42554013993311 | -0.74872260562074 |
| S | -0.06193106691959 | -2.08597697723231 | 2.64034015131627 |

## 10.2 Molecular orbitals obtained from the CSF-ROHF calculation

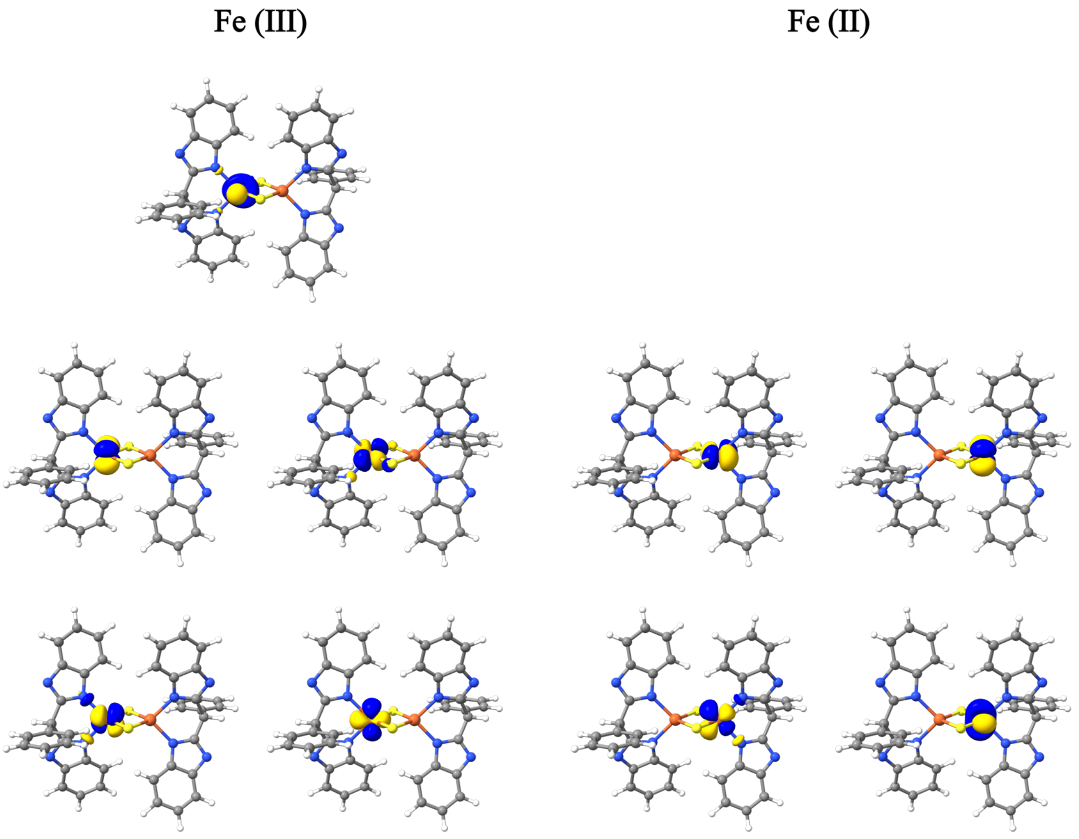


Figure S7: Canonical molecular orbitals obtained from the CSF-ROHF calculation of $[L_2Fe^{II,III}_2S_2]^{3-}$ in the $S = \frac{1}{2}$ spin state.

## 10.3 GS-ROCIS Calculated $L_{2,3}$-edge XAS

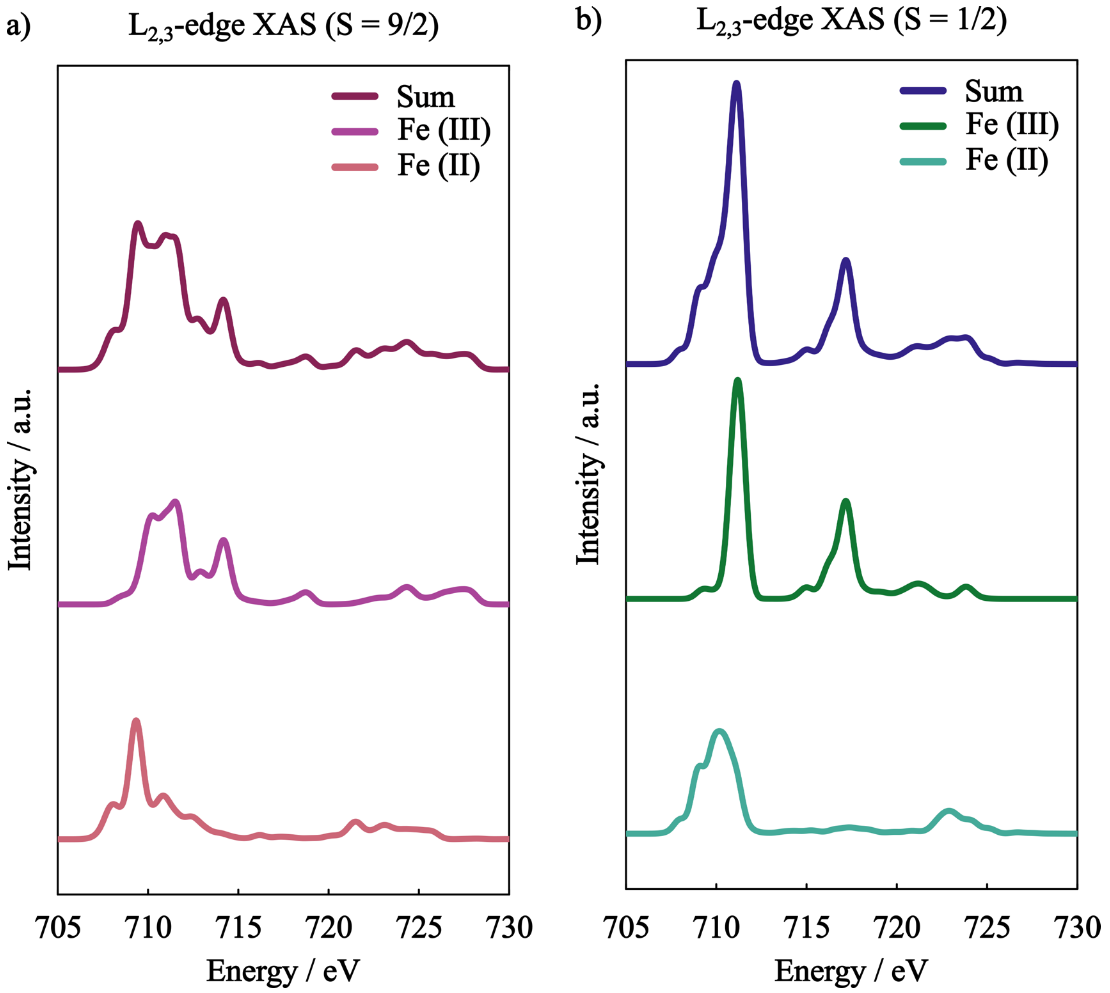


Figure S8: GS-ROCIS calculated $L_{2,3}$-edge absorption spectra of $[L_2Fe^{II,III}{}_2S_2]^{3-}$ with a) ferromagnetic and b) antiferromagnetic coupling between the Fe centers.

## 11 References

(1) Piligkos, S.; Slep, L. D.; Weyhermüller, T.; Chaudhuri, P.; Bill, E.; Neese, F. Magnetic Circular Dichroism Spectroscopy of Weakly Exchange Coupled Transition Metal Dimers: A Model Study. *Coord. Chem. Rev.* **2009**, *253* (19), 2352–2362. https://doi.org/10.1016/j.ccr.2008.10.014.
